# The role of the WD40-repeat protein family in cancer

**DOI:** 10.1186/s12943-026-02718-2

**Published:** 2026-06-27

**Authors:** Jiale Li, Haixu Zhou, Qisheng Luo, Duorong Wu, Maximo Lin, Ge Mai, Xindong Xu, Yongping He, Yanhong Liu, Jiachong Wang, Changfeng Miao, Zigui Chen, Chunhai Tang

**Affiliations:** 1 Department of Cerebrovascular Diseases, Haikou Affiliated Hospital of Central South University Xiangya School of Medicine, Haikou, 570208 China; 2 Department of Neurosurgery, Yantai Affiliated Hospital of Shandong Medical and Pharmaceutical University, Yantai, Shandong 264003 China; 3 Department of Neurosurgery, Affiliated Hospital of Youjiang Medical University for Nationalities, Baise, Guangxi 533000 China; 4 Guangxi Engineering Research Center for Biomaterials in Bone and Joint Degenerative Diseases, Baise, Guangxi 533000 China; 5 Department of Clinical Laboratory, The Third People’s Hospital of Haikou City, Haikou, 570208 China; 6 Department of Neurosurgery Second Branche, Hunan Provincial People’s Hospital (The First Affiliated Hospital of Hunan Normal University), Changsha, Hunan 410005 China; 7 Department of Neurosurgery, The Second Affiliated Hospital of Guangxi Medical University, Nanning, Guangxi 530005 China

## Abstract

WD40-repeat (WDR) proteins constitute one of the largest and most functionally diverse scaffold families in eukaryotes. By folding tandem WD repeats into β-propeller domains, they provide modular interaction surfaces that assemble multi-protein complexes governing transcription and epigenetic control, ubiquitin-dependent proteostasis, RNA metabolism, and cell-cycle progression. Despite their pervasive involvement in oncogenic signaling and hallmark cancer phenotypes, the cancer field still lacks an integrated framework that connects WDR structural logic to context-dependent mechanisms and, critically, to actionable biomarkers and therapeutic strategies; existing evidence remains dispersed across tumor types and molecular pathways. This review synthesizes current knowledge to address that gap. We first summarize core structural principles of WD40 β-propellers and explain how multivalent binding and partner selection enable WDR proteins to function as assembly platforms in oncogenic networks. We then consolidate mechanistic evidence showing how representative WDR proteins shape malignant state transitions, including sustained proliferation, survival under stress, epigenetic plasticity, invasion and metastasis, and therapy resistance, by rewiring chromatin programs, ubiquitination circuits, and RNA and translation outputs across cancers. Finally, we highlight translational progress and opportunities. Overall, this review integrates the fragmented WDR research into a clinically oriented framework that clarifies their potential as biomarkers for early detection, stratified diagnosis, and treatment-response prediction, and delineates druggable entry points and rational combination strategies, thereby providing a translational roadmap to enable more precise cancer diagnosis and more effective targeted therapies in the future.

**Supplementary Information:**

The online version contains supplementary material available at https://doi.org/10.1186/s12943-026-02718-2.

## Introduction

WDR proteins constitute one of the largest and most functionally diverse protein families in eukaryotes, characterized by the presence of 40–60 amino acid repeats ending with tryptophan-aspartic acid (W-D) dipeptides. These repeats typically fold into a β-propeller structure that serves as a modular platform for protein–protein interactions, enabling WDR proteins to participate in a wide array of biological processes including transcriptional regulation, epigenetic modification, ubiquitination, and cell cycle control [1–3].

In the context of human disease growing evidence implicates WDR proteins in oncogenesis, with context-dependent oncogenic or tumor-suppressive roles. WDR5, a core subunit of COMPASS histone H3K4 methyltransferase complexes, promotes H3K4 methylation and cooperates with MYC by facilitating MYC recruitment to chromatin and activation of MYC-driven transcriptional programs [4, 5]. Conversely, the WD40 F-box protein FBXW7 acts as a tumor suppressor by serving as an SCF substrate receptor that targets Cyclin E, c-MYC, and NOTCH for ubiquitin-mediated proteasomal degradation [6]. Moreover, members of the WDR family are deeply integrated into hallmark cancer pathways, including PI3K/AKT [7], Wnt/β-catenin [8], and NF-κB [9, 10] signaling cascades. Their influence extends to key cancer hallmarks such as sustained proliferation, evasion of apoptosis, invasion and metastasis, and therapy resistance. As high-throughput genomic and proteomic profiling expands, WDR genes are increasingly recognized as potential biomarkers for cancer diagnosis, prognosis, and therapeutic response [11, 12].

Despite the biological and clinical significance of WDR proteins, current evidence remains fragmented across tumor types, molecular pathways, and therapeutic contexts. This review therefore aims to provide an integrated framework for understanding how WDR proteins contribute to cancer biology and clinical translation. Rather than treating WDR proteins as an isolated catalogue of cancer-associated factors, we organize this review around a structure–module–state framework. First, we define how recurrent structural features of WD40 β-propellers, including top-face pockets, rim-loop interfaces, degron-recognition surfaces, reader-like binding pockets, and multivalent scaffold regions, generate distinct modes of molecular recognition. Second, we map these interaction modes onto recurrent cancer-relevant functional modules, including chromatin and epigenetic regulation, ubiquitin-dependent proteostasis, RNA and translation control, autophagy and membrane trafficking, and cytoskeletal signaling. Finally, we evaluate how these modules create tumor-state-specific dependencies that can be translated into diagnostic or prognostic biomarkers, predictive markers of treatment response, druggable interfaces, targeted degradation strategies, and rational combination therapies. Through this framework, the review connects WDR structural logic with oncogenic mechanism and clinical actionability.

## Structural and functional characteristics of WDR proteins

### WD40 motif: structure, folding, and evolution

**Fig. 1:**
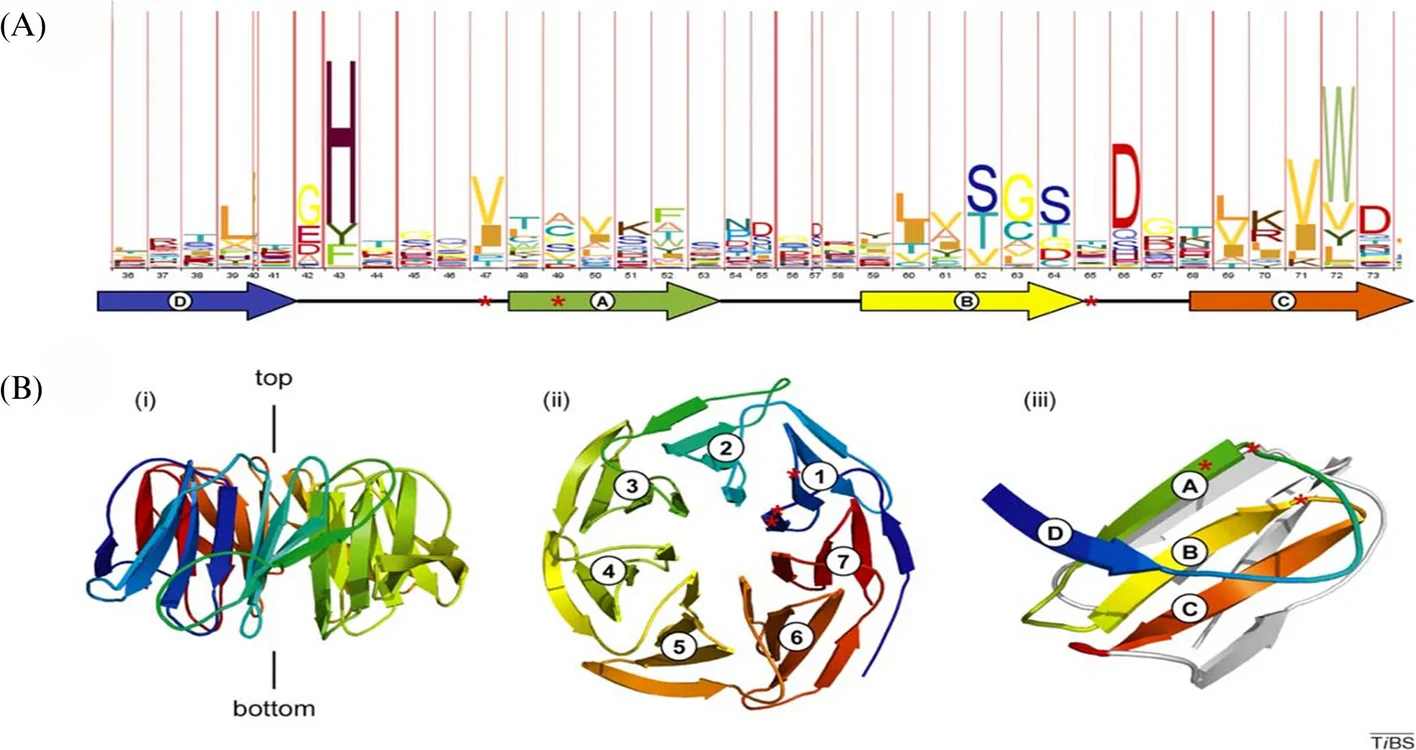
Sequence and structural features of WD40 domains, which contain several copies of 44–60 residue WD40 repeats. WD40 repeats typically fold into seven-bladed β-propellers with each blade comprising a four-stranded anti-parallel β-sheet. **A** Sequence logo for WD40 repeats. The letter plots represent amino acid conservation at each position. The corresponding β-strands within a repeat are depicted below. Each WD40 repeat typically contains a variable region of 11–24 residues followed by key signature sequence features: a glycine-histidine (GH) dipeptide at the end of strand D, three small amino acids (serine, glycine etc.) at the C-terminal end of strand B and a tryptophan-aspartate (WD) dipeptide at the end of strand C. The logo was created with HMMlogo based on a structural alignment of WD40 domains as classified in SCOP (PDB IDs 1tbg, 1erj, 2ce8, 1u2v, 1nex, 1p22, 1nr0, 1pi6,1sq9, 1vyh, 2ovr and 1yfq). **B** WD40 domains often fold into seven-bladed β-propellers with a funnel-like shape and, by convention, the narrower part of the funnel containing the DA and BC loops is defined as the top domain and the wider part as the bottom of the propeller (i). The WD40 sequence repeat corresponds to strand D of one blade followed by strands A, B and C of the next blade (iii). The WD40 sequence repeat therefore is not equivalent to a single propeller blade. The main chain is coloured using a gradient from red at the N-terminus to blue at the C-terminus (ii, iii). Residues often involved in peptide interactions on the top surface are highlighted with red asterisks. Reproduced from ref.15 with permission from Elsevier 2010

The WD40 (WD-repeat) motif was originally defined as a ~ 40–60 amino-acid repeat that frequently contains an N-terminal GH dipeptide and terminates with a WD (Trp-Asp) dipeptide, although many bona fide WD repeats deviate from this consensus and show low overall sequence conservation [13, 14]. Despite sequence variability, WD repeats converge on a highly conserved 3D architecture: tandem repeats assemble into a β-propeller domain, most commonly a seven-bladed propeller, where each blade is typically composed of four antiparallel β-strands [15] (Fig. 1). High-resolution structures of heterotrimeric G proteins established the stereochemical basis of the WD repeat fold. In the Giα1β1γ2 heterotrimer and in the transducin βγ dimer, the Gβ subunit forms a circularized sevenfold β-propeller built from WD repeats, providing a structural paradigm for WD40 domains broadly [16, 17]. A hallmark of many WD40 propellers is the “Velcro closure”: terminal strands from the N- and C-termini pair to “snap” the ring shut, stabilizing the propeller topology and reducing end-fraying. A hallmark of many WD40 propellers is the “Velcro closure”: terminal strands from the N- and C-termini pair to “snap” the ring shut, stabilizing the propeller topology and reducing end-fraying [18, 19]. Functionally, WD40 domains are generally non-enzymatic and act as interaction platforms—their top face, bottom face, and perimeter loops provide chemically diverse surfaces that support assembly of multi-protein complexes and transient regulatory interactions [15, 20]. WD40 propellers exemplify a common principle of repeat proteins: despite low overall sequence conservation, the β-propeller topology is strongly constrained [21]. Substantial sequence divergence is tolerated provided β-strand-forming patterns and the inter-strand backbone hydrogen-bonding framework (and compatible core packing/signature positions) are maintained [22, 23]. Experimental mutagenesis and folding analyses, particularly in canonical WD-repeat proteins such as Gβ and Sec13, show that conserved Asp residues within WD repeats are important but not uniformly essential; different Asp positions contribute unequally to folding efficiency and local stability, and some effects appear to reflect altered folding pathways or dependence on cellular folding machinery (e.g., chaperonins) [24, 25]. The Velcro closure further contributes to robustness by coupling terminal segments into the propeller’s hydrogen-bond network, which helps explain why WD40 domains often behave as rigid scaffolds once folded [18, 19].

Early comparative analyses emphasized WD-repeat proteins as an “ancient regulatory” family that is abundant in eukaryotes and involved in diverse processes, consistent with their role as versatile scaffolds [14]. As genome data expanded, it became clear that WD40 domains also exist in prokaryotes: systematic surveys identified thousands of bacterial/archaeal WD40 proteins and highlighted both similarities (β-propeller architecture) and distinctive features (e.g., highly repetitive WD40 proteins) relative to eukaryotic counterparts [26]. Mechanistically, WD40 evolution is well explained by repeat duplication, divergence, and domain shuffling: adding or modifying blades can tune surface chemistry and binding specificity without disrupting the core fold, enabling rapid functional diversification while retaining a stable interaction platform [27]. In parallel, resources and annotation frameworks (e.g., domain databases and WD40-focused datasets) reflect the practical challenge that WD repeats are often difficult to detect purely by primary sequence, reinforcing the need to integrate secondary-structure/propeller-aware models into evolutionary and functional annotation [28].

### General functional roles

#### Scaffold platforms for complex assembly

One of the primary functions of WDR proteins is their role as protein interaction hubs, where they serve as platforms for assembling multi-protein complexes. Due to their versatile structural architecture, WD40 domains can mediate a wide array of protein–protein interactions through their top face and perimeter loops. The β-propeller structure creates a large, flat surface that can accommodate multiple interaction partners, which is essential for coordinating complex cellular processes. WD40 proteins often function as scaffolds, bringing together various signaling molecules, enzymes, and structural proteins to facilitate cellular responses to environmental signals, metabolic changes, and developmental cues [2, 29]. This interaction capability is especially important in signal transduction pathways, where WD40 domains often associate with proteins that regulate cellular signaling, transcription, and metabolism. For example, the WD40 domain in F-box proteins serves as a substrate receptor for the SCF (Skp1-Cullin-F-box) ubiquitin ligase complex, influencing the degradation of key cell cycle regulators and other substrates [30]. Similarly, the β-transducin repeat containing proteins (β-TrCP) participate in the Wnt/β-catenin signaling pathway, affecting gene transcription. By organizing large complexes, WDR proteins enable cells to efficiently process signals and coordinate a broad range of biological functions [31, 32].

#### Substrate adaptors in ubiquitination

WDR proteins commonly act as substrate adaptors that confer target specificity to ubiquitination machineries, translating upstream signaling into selective proteostasis outputs. In the cullin–RING ligase (CRL) superfamily, substrate selection is largely delegated to exchangeable receptors/adaptors, many of which deploy WD40 β-propellers to recognize short degron motifs (often phosphorylation-dependent) and present substrates to the catalytic core for ubiquitin transfer [33]. A canonical example is the SCF (SKP1–CUL1–F-box) system, where WD40-containing F-box receptors such as β-TrCP bind phosphodegrons to drive signal-coupled degradation programs in pathways including NF-κB and Wnt/β-catenin [34]. In parallel, CRL4 (CUL4–DDB1) ligases use DDB1 as a linker to recruit a large repertoire of WD40 substrate receptors (DCAFs/DWD proteins), greatly expanding substrate space and enabling context-specific ubiquitination across chromatin- and genome-regulatory processes relevant to cancer and cell-state control [35]. Beyond CRLs, the anaphase-promoting complex/cyclosome (APC/C) relies on WD40-containing coactivators CDC20 and CDH1 as substrate adaptors to recognize destruction motifs and enforce ordered proteolysis during cell-cycle transitions, a mechanism increasingly refined by recent structural and mechanistic analyses [36].

#### Chromatin-anchored recruitment and functional modulation of epigenetic complexes

WDR proteins are widely used as chromatin-anchoring modules that position epigenetic machineries on nucleosomes and tune their catalytic output in a context-dependent manner. Mechanistically, the β-propeller provides a stable interaction surface,often via the top face and peripheral loops,that can engage histone tails, histone marks, or partner subunits to promote locus-specific assembly and allosteric regulation of chromatin complexes. A well-established paradigm is the PRC2 subunit EED, whose WD40 domain contains an aromatic cage that recognizes pre-existing H3K27me3 and transduces this binding event into allosteric activation of PRC2 methyltransferase activity, reinforcing Polycomb repression [37]. In parallel, WDR5 functions as a chromatin-facing scaffold within SET1/MLL (COMPASS-like) complexes, coordinating protein and RNA contacts that support H3K4 methylation programs and transcriptional state maintenance, and recent work continues to refine how its interaction surfaces (e.g., the WIN site) can anchor regulatory assemblies with therapeutic implications [38]. Finally, histone-binding WD40 proteins RBBP4/7 (RbAp46/48) act as docking/reader-like components across multiple chromatin-modifying and remodeling complexes (including NuRD and PRC2-associated assemblies), where they bind H3/H4 regions and help stabilize complex–nucleosome engagement, thereby shaping chromatin accessibility and transcriptional outputs in development and cancer [39].

#### RNA-Centric hubs in transcript processing and ribosome biogenesis

WDR proteins are also important organizers of RNA-centered processes, including transcript maturation, RNA modification, splicing, translation control, and ribosome biogenesis. Unlike chromatin-associated WDR proteins, which mainly influence transcriptional programs, or ubiquitin-ligase adaptors, which regulate protein turnover, RNA-related WDR proteins act at an intermediate step between gene transcription and protein production. They help determine how nascent transcripts are processed, modified, selected for translation, and ultimately converted into functional proteomes. This level of regulation is highly relevant to cancer biology, because malignant cells frequently rely on efficient mRNA maturation, altered isoform usage, selective translation, and increased ribosome production to support proliferation, cellular plasticity, and adaptation to therapeutic stress [40, 41].

WDR33 provides a representative example of this function in pre-mRNA 3′-end processing. As a core component of the cleavage and polyadenylation specificity factor complex, WDR33 cooperates with CPSF30 to recognize the canonical AAUAAA polyadenylation signal and contributes to accurate pre-mRNA 3′-end formation [42, 43]. This process is not merely a constitutive housekeeping event. Changes in polyadenylation site selection can reshape 3′UTR length, RNA stability, miRNA accessibility, RNA-binding protein recruitment, and translational efficiency [44]. Therefore, WDR33-associated 3′-end processing may contribute to oncogenic transcriptome remodeling by influencing which mRNA isoforms are stabilized and translated in tumor cells. RNA modification and translation control represent another important dimension of this module. The METTL1–WDR4 complex is a well-defined example, in which WDR4 acts as the WD40 cofactor of METTL1-mediated N^7^-methylguanosine modification of tRNAs. Through this mechanism, WDR4 can influence tRNA stability, codon decoding, and the selective translation of transcripts associated with growth, invasion, and stress adaptation [45]. This mechanism has been linked to several cancer contexts, including lung cancer progression [45], nasopharyngeal carcinoma chemoresistance [46], and esophageal squamous cell carcinoma tumorigenesis [47]. Thus, WDR4 connects the scaffold function of WD40 proteins with epitranscriptomic regulation and oncogenic proteome remodeling. Splicing-associated WDR proteins further broaden the role of this RNA-centered network. MEP50/WDR77, the WD40 cofactor of PRMT5, supports PRMT5-containing methyltransferase complexes and has been implicated in alternative splicing, transcriptional regulation, and invasion-related programs [48, 49]. Rather than acting as a simple accessory subunit, WDR77 helps maintain and specify PRMT5 complex activity, thereby linking arginine methylation to RNA processing and malignant cell-state regulation. Nucleolar WDR proteins connect RNA metabolism with ribosome production and translational capacity. WDR12, a component of the PES1–BOP1–WDR12 PeBoW complex [50], supports large ribosomal subunit maturation, whereas WDR43/Utp5 participates in early pre-rRNA processing and small ribosomal subunit biogenesis [51]. These proteins may be particularly important in tumor states marked by MYC activation, rapid biomass accumulation, stem-like properties, or treatment-induced stress, where ribosome output can become a limiting factor for continued growth [52]. For example, WDR12 is required for ribosome biogenesis in glioma stem-like cells, while WDR43 has been linked not only to ribosome biogenesis but also to chromatin-associated RNA binding and Pol II transcriptional output [40, 50]. Taken together, RNA-processing and ribosome-biogenesis WDR proteins form an RNA-to-proteome regulatory axis that complements chromatin-associated WDR proteins controlling transcriptional input and ubiquitin-associated WDR proteins controlling protein turnover.

#### Membrane trafficking and autophagy

WDR proteins also operate as membrane-localized adaptors that decode phosphoinositide signals and organize trafficking/autophagy assemblies at specific membrane subdomains. A central paradigm is the PROPPIN/WIPI family (β-propellers that bind phosphoinositides), whose WD40 β-propeller fold engages PI3P-enriched membranes via conserved binding motifs and thereby functions as a spatial “landing pad” for downstream effector recruitment during autophagosome biogenesis [53]. In mammals, WIPI2 exemplifies this adaptor logic by coupling PI3P-positive phagophore membranes to the LC3 lipidation machinery through recruitment and engagement of the ATG12–ATG5–ATG16L1 complex. WIPI4 (WDR45) acts at later stages by coordinating with factors such as ATG2 to support membrane expansion and sustain autophagic flux, and these mechanisms continue to be refined by recent cellular and structural studies, including disease-relevant analyses of WDR45/WIPI4 dysfunction [54, 55]. Beyond canonical autophagy, PROPPINs are now also discussed as broader endomembrane organizers that can influence endo-lysosomal remodeling (including membrane fission behaviors), reinforcing the view that WD40 propellers can serve as versatile phosphoinositide-directed adaptors connecting membrane identity to dynamic trafficking outputs [56].

#### Cytoskeleton and polarity couplers linking structure to signal transduction

WDR proteins can act as cytoskeleton and polarity couplers by positioning signaling enzymes and adaptors on structural platforms such as focal adhesions, cortical actin networks, and polarity-associated assemblies, thereby converting changes in cell architecture and mechanical context into tuned signal-transduction outputs. A prominent example is RACK1 (GNB2L1), a seven-bladed WD40 scaffold that integrates integrin and focal-adhesion signaling with actin–spectrin organization by assembling membrane-proximal complexes containing kinases and adaptors (e.g., FAK/Src/AKT modules), supporting coordinated adhesion dynamics and downstream signaling [57]. In parallel, WD40 control of actin turnover provides a direct route to coupling structure to signaling: WDR1/AIP1 promotes rapid cofilin-driven actin filament disassembly, a mechanism sharpened by recent structural and mechanistic work that explains how accelerated filament dismantling can reprogram cortical tension and protrusive behavior, with clear relevance to cell motility and immune-cell function [58]. At the level of polarity and pathway routing, the striatin (STRN) family serves as WD40-containing scaffolds within STRIPAK signaling assemblies, which are increasingly recognized as hubs that coordinate kinase/phosphatase signaling with cytoskeletal organization and polarized cellular behaviors [59].

### Classification and family members

WDR proteins are unified by a conserved β-propeller fold assembled from degenerate WD repeats, yet they display remarkable sequence divergence and extensive architectural diversity. As a result, the field has converged on structure- and context-aware classification strategies rather than sequence-only schemes. Authoritative structural syntheses emphasize that the β-propeller provides reusable interaction surfaces (top face, rim loops, and—in many proteins—a central pocket/channel), enabling WDR proteins to function as modular interaction “hardware” across signaling, chromatin regulation, and proteostasis [15, 20]. This modularity also underlies the growing “target class” view of WDR domains, supported by chemical probe development against prototypic members such as WDR5 and EED [2, 60, 61].

#### Domain-architecture–based classification

**Fig. 2:**
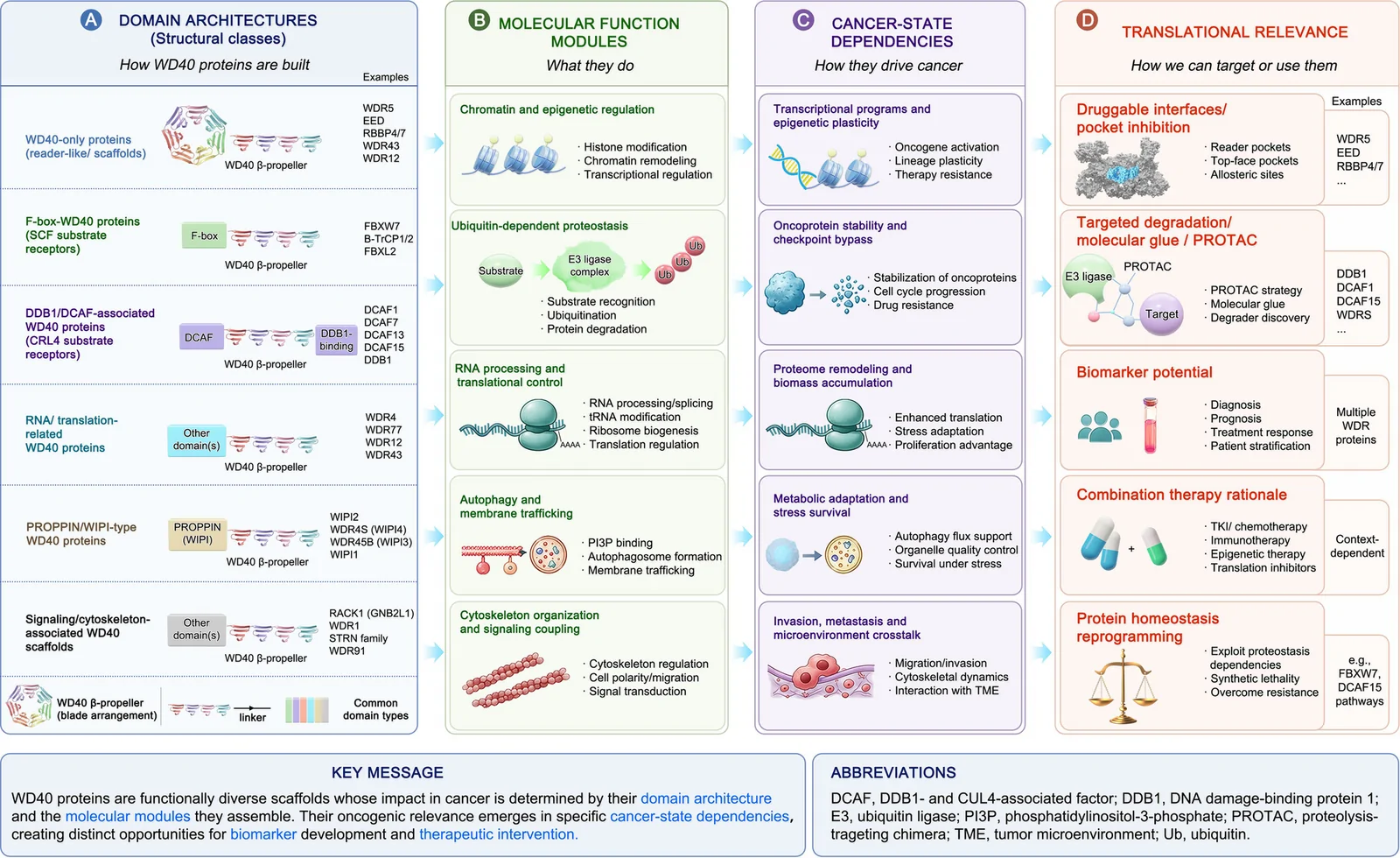
Domain architecture-based and function-oriented classification of cancer-relevant WD40 proteins. This schematic links WD40 protein architecture to molecular function, cancer-state dependency, and translational relevance. **A** Cancer-related WD40 proteins are classified by structural features and complex-association patterns, including WD40-only scaffolds, F-box–WD40 proteins, CRL4-DDB1-DCAF-associated proteins, RNA/translation-related WD40 proteins, PROPPIN/WIPI-type proteins, and signaling/cytoskeleton-associated scaffolds. **B** These proteins are reorganized into functional modules involving chromatin regulation, ubiquitin-dependent proteostasis, RNA processing and translation, autophagy/membrane trafficking, and cytoskeleton-linked signaling. **C** These modules contribute to malignant phenotypes such as transcriptional plasticity, oncoprotein stability, stress adaptation, invasion, metastasis, and tumor–microenvironment crosstalk. **D** The framework highlights potential translational opportunities, including pocket inhibition, targeted degradation, biomarker development, rational combination therapy, and proteostasis-based intervention. Overall, the figure emphasizes that the cancer relevance of WD40 proteins depends on the integration of domain architecture, molecular module, and tumor context

A particularly explicit and widely citable framework comes from a systematic survey of the human WD40 proteome. Zou and colleagues identified 262 non-redundant human WD40 proteins and grouped them into 21 classes based on domain architecture, separating “WD40-only” proteins from multi-domain proteins in which WD40 propellers are paired with additional modules (e.g., F-box, LisH, bromodomains, SOCS box, BEACH-like regions) [29] (Fig. 2). This architecture-centric taxonomy is attractive for cancer reviews because it aligns with how WDR proteins implement function: domain combinations often predict which macromolecular machine a WDR protein participates in (e.g., E3 ligases vs chromatin complexes), thereby providing an interpretable bridge from proteome catalogs to mechanism.

#### Module- and complex-centric classification

Complementary to architecture-based grouping, a second widely used approach classifies WDR proteins by molecular module membership, reflecting the recurring roles of β-propellers as substrate receptors, scaffolds, and adaptors within canonical cellular machines [62, 63]. In practice, this module-centric view has been particularly well formalized in systems where WD40 domains provide specificity determinants, particularly ubiquitin ligases [35, 64] and chromatin complexes [65], and it maps naturally onto cancer-relevant dependencies.

##### WD40 substrate receptors/adaptors in ubiquitin ligase systems

A large fraction of oncologically important WD40 proteins function as substrate-recognition subunits for E3 ligases. In SCF complexes, mammalian F-box proteins are formally organized into FBXW, FBXL, and FBXO subfamilies based on their substrate-binding domains, with FBXW denoting F-box proteins carrying WD40 repeats (e.g., FBXW7 and β-TrCP) [66, 67]. In CRL4 complexes, DDB1 serves as a linker that recruits WD40 substrate receptors. He et al. defined a DWD box (DDB1-binding WD40 protein motif) and experimentally demonstrated DDB1 association for multiple DWD proteins, establishing a concrete rule set for identifying this WD40 receptor class [68]. CUL4–DDB1 complexes engage a broad repertoire of WD40 repeat‑containing substrate receptors, as demonstrated by early work showing interactions with WDR5 and EED, core components of histone methylation complexes. Recent interactomic analyses further expand the landscape of DDB1‑associated WD40 proteins, identifying dozens of high‑confidence DCAF candidates that link CRL4 to diverse biological pathways [69]. Beyond serving as substrate receptors, β‑propeller WD40 domains can also function as ubiquitin‑binding modules. Seminal work demonstrated that ubiquitin recognition by the β‑propeller of F‑box adaptors such as Cdc4/Fbw7 promotes their autoubiquitination and proteasomal turnover, highlighting a mechanism by which WD40 domains act as regulatory elements within proteostasis circuits [70].

##### Chromatin and epigenetic modules

A second high-impact module class comprises WD40 proteins embedded in chromatin regulatory machinery, in which β-propeller folds provide multivalent interaction surfaces to assemble and stabilize epigenetic complexes and, in some cases, engage histone-tail features [71, 72]. WDR5 is a core component of SET1/MLL H3K4 methyltransferase assemblies (WRAD module) and a leading paradigm for drugging WD40-mediated protein–protein interactions [73, 74]. Chemical probes targeting the WDR5 WIN site, exemplified by OICR-9429, disrupt WDR5–MLL1 engagement and rewire WDR5-dependent transcriptional programs with anti-tumor effects in multiple cancer contexts [61]. More recent WIN-site inhibitors and WDR5 degraders further demonstrate rapid chromatin displacement of WDR5 and potent oncogenic pathway suppression, refining the mechanistic framework for therapeutic targeting of this WD40 hub [75, 76].

##### Transcriptional coregulator and signaling-coupler adaptors

A third recurrent module class includes WD40-containing adaptors that couple transcriptional regulatory complexes to pathway outputs. TBL1X and TBL1XR1 (TBLR1) are F-box/WDR proteins in these complexes, where their multivalent interaction surfaces support corepressor engagement and promoter-bound complex remodeling. Mechanistic work established that TBL1/TBLR1 can act as adaptors for recruiting ubiquitin-conjugating activity and the 19S proteasome, enabling ligand-dependent exchange of NCoR/SMRT corepressors for coactivators and thereby coupling transcriptional states to pathway activation [77, 78]. In cancer-relevant contexts, TBL1X/TBL1XR1 have been repeatedly positioned at the intersection of major transcriptional programs, including Wnt/β-catenin and NF-κB signaling, consistent with a role as pathway-to-transcription coupling nodes [79]. Importantly, tumor genomics provides orthogonal support for this adaptor paradigm: TBL1XR1 harbors recurrent somatic alterations in aggressive B-cell lymphomas, including functionally implicated mutations and recurrent fusions such as TBL1XR1/TP63, and additional leukemia-associated fusions continue to expand its oncogenic repertoire [80, 81]. These convergent mechanistic and genetic lines of evidence motivate treating TBL1X/TBL1XR1 as a distinct, cancer-relevant WD40 coregulator/adaptor module with emerging druggability.

#### Phylogenetic/topology and resource-driven subtyping

In addition to proteome-scale domain-architecture classes, WD40/WDR annotation is increasingly refined using phylogenetic/similarity clustering within defined datasets, β-propeller topology (blade number and tandem/multi-propeller organization), and structure-informed resources, which together improve candidate prioritization when WD motifs are sequence-degenerate. Topology matters because β-propellers show substantial architectural diversity, including oligomeric and atypical assemblies [82, 83], and disease-relevant tandem propellers exemplified by the EML1 “TAPE” domain, where one blade is formed from discontinuous sequence segments [84]. Resource-wise, WDSPdb 2.0 provides WD40-focused predicted secondary/tertiary structures and featured-site (hotspot) annotations that enable fold-aware comparisons beyond motif scans [62], while major infrastructure increasingly embeds structure at scale: Pfam (2025) introduced new entries designed to capture the entire WD40 β-propeller for more specific annotation [85], InterPro (2025) links family/domain assignments to PDBe and AlphaFold DB across > 200 million sequences [86], and UniProt (2025) continues shifting toward high-quality, non-redundant reference-proteome–centered pipelines [87].

### A structure–module–state framework for interpreting WDR function and actionability in cancer

**Fig. 3:**
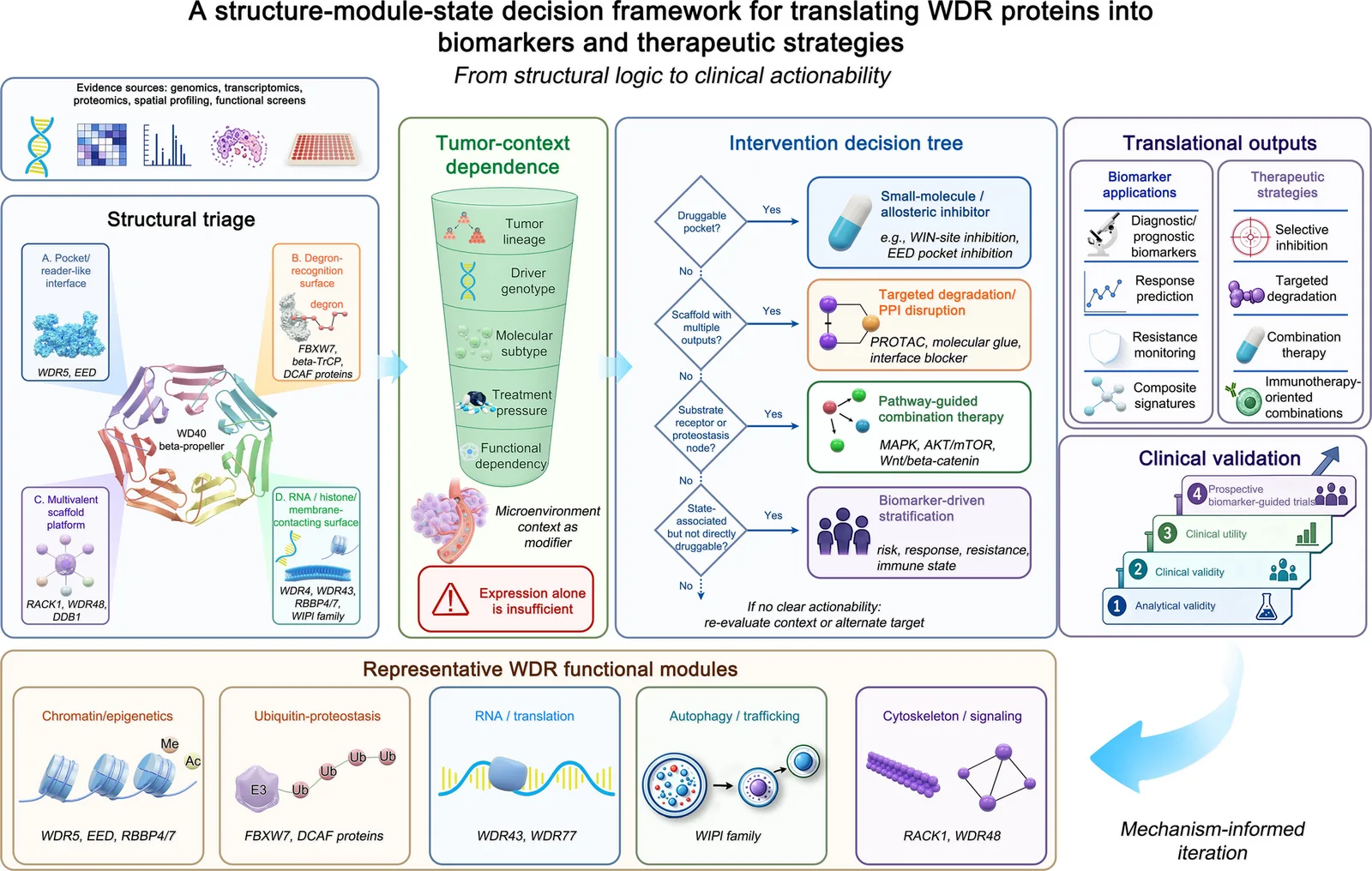
Structure–module–state decision framework for translating WDR proteins. This schematic presents a framework for evaluating the translational actionability of cancer-associated WDR proteins. Firstly, candidate WDR proteins are identified from multi-omics, spatial profiling, and functional screening datasets, followed by structural triage of actionable features such as druggable pockets, degron-recognition surfaces, scaffold platforms, and RNA-, histone-, or membrane-contacting interfaces. Secondly, tumor-context dependence is assessed by integrating tumor lineage, driver genotype, molecular subtype, treatment pressure, functional dependency, and microenvironmental modifiers, emphasizing that expression alone is insufficient. Then, these structural and contextual features are linked to intervention strategies, including small-molecule inhibition, targeted degradation, protein–protein interaction disruption, pathway-guided combination therapy, and biomarker-driven stratification. Finally, translational outputs are evaluated through biomarker applications, therapeutic strategies, and progressive clinical validation from analytical validity to prospective biomarker-guided trials. Representative WDR functional modules are shown at the bottom to support mechanism-informed refinement

To provide a conceptual framework for the following sections, we propose that cancer-associated WDR proteins can be interpreted through a structure–module–state model. In this model, the β-propeller architecture first determines the mode of molecular recognition, including top-face pockets, rim-loop interfaces, degron-binding grooves, histone- or RNA-contacting surfaces, phosphoinositide-binding motifs, and multivalent partner-binding platforms [20, 88, 89]. These structural features then place individual WDR proteins into functional modules, such as chromatin and epigenetic scaffolds, ubiquitin-ligase substrate receptors, RNA and translation hubs, autophagy and membrane-trafficking adaptors, or cytoskeleton-associated signaling couplers [90, 91]. The oncogenic consequence of each WDR protein is therefore not defined by the WD40 fold alone, but by the tumor-state dependency created when a specific structural interface is embedded within a lineage-, genotype-, or treatment-specific regulatory circuit. This framework also helps prioritize clinical translation: pocket-containing or reader-like WDR proteins, such as WDR5 and EED, are more amenable to small-molecule or allosteric inhibition [92, 93]; scaffold-like WDR proteins with multiple outputs may be better suited to targeted degradation or interface-disrupting strategies; and substrate-receptor WDR proteins may serve as biomarkers of proteostatic rewiring or guide downstream pathway-based combination therapy [94, 95]. Importantly, WDR proteins should not be interpreted simply as generic components of oncogenic pathways. Their functional impact depends on their precise mechanistic position within a given signaling circuit. In some settings, WDR proteins act proximally as scaffolds or adaptors that assemble signaling complexes; in others, they serve as regulatory nodes that tune pathway intensity through ubiquitination, deubiquitination, chromatin recruitment, RNA modification, or protein-stability control. In still other contexts, they mainly amplify downstream outputs such as transcription, translation, ribosome biogenesis, autophagy, or cytoskeletal remodeling. Therefore, throughout this review, we distinguish whether a given WDR protein functions as a complex-organizing scaffold, a pathway regulator, or an output-level effector in each tumor context (Fig. 3).

## WDR Proteins in tumor

**Fig. 4:**
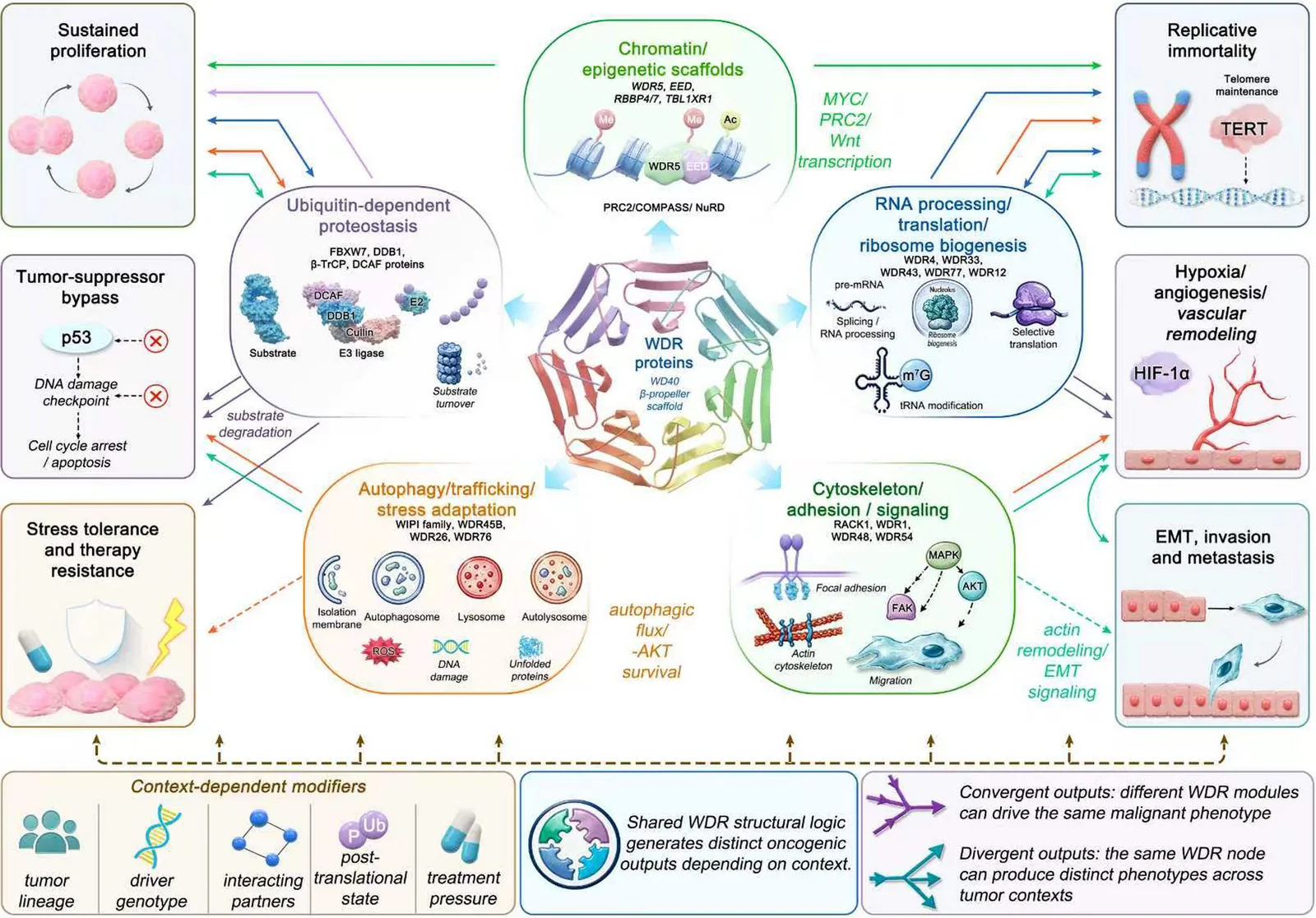
Convergent and divergent WDR-dependent mechanisms across cancer hallmarks. This schematic illustrates how cancer-associated WDR proteins function as WD40 β-propeller scaffolds to organize distinct molecular modules and drive tumor phenotypes. At the center, WDR proteins serve as interaction platforms that connect chromatin/epigenetic regulation, ubiquitin-dependent proteostasis, RNA processing/translation/ribosome biogenesis, autophagy and trafficking, and cytoskeleton/adhesion signaling. These modules converge on major cancer hallmarks, including sustained proliferation, tumor-suppressor bypass, stress tolerance and therapy resistance, replicative immortality, hypoxia/angiogenesis/vascular remodeling, and EMT, invasion, and metastasis. The biological outputs of WDR proteins are further shaped by tumor lineage, driver genotype, interacting partners, post-translational modifications, and treatment pressure. Colored arrows indicate that different WDR modules can converge on similar malignant phenotypes, whereas the same WDR node may produce divergent outcomes across tumor contexts

With the rapid progress of research on WDR proteins in recent years, accumulating evidence indicates that, by engaging and integrating multiple key oncogenic pathways, these proteins play important regulatory roles in tumor initiation, progression, and malignant evolution (Fig. 4).

### Mitogenic-to-transcriptional coupling for sustained proliferation

A core requirement for sustained proliferation is not merely transient activation of upstream mitogenic cascades, but the locking-in of durable transcriptional and proteostatic outputs that continuously support biomass accumulation, cell-cycle progression, and growth-factor independence. WDR proteins are well suited to this role because β-propeller scaffolds can stabilize multi-component assemblies, sharpen pathway coupling, and bias gene-expression programs toward proliferative states [96, 97].

WDR5 provides a paradigmatic mechanism by which oncogenic signaling is converted into persistent transcriptional output. Mechanistic and structural studies demonstrate that MYC engages WDR5 as a chromatin-associated cofactor, and that this interaction is required for efficient recruitment of MYC to specific growth-associated loci, thereby sustaining a high-output proliferative transcriptional state even when upstream cues fluctuate [4, 98]. Beyond serving as a MYC cofactor, WDR5 also functions as a broader chromatin-tethering hub via the WIN site, a pocket that mediates interactions with SET1/MLL family complexes and supports WDR5 residence on chromatin. Pharmacologic or genetic disruption of the WIN site rapidly displaces WDR5 from chromatin and rewires WDR5-dependent gene networks, consistent with a model in which WDR5 “licenses” mitogenic transcription by stabilizing epigenetic/transcriptional assemblies that maintain anabolic and cell-growth programs [61, 92].

In contrast to WDR5’s chromatin-licensing role, DCAF7/WDR68 illustrates how a WD40 scaffold can increase the efficiency of signal transmission from kinase modules to downstream growth phenotypes. The WDR protein HAN11 (DCAF7/WDR68) was originally established as a scaffold coordinating MEKK1–HIPK2 signaling, providing a direct molecular bridge between kinase inputs and effector outputs [99, 100]. More recently, DCAF7 has been shown to act as a scaffold for IRS1–PI3K, supporting efficient AKT activation and FOXO1 inhibition, and thereby promoting cell-cycle progression and proliferation—an architecture that tumor cells can co-opt to sustain growth/survival signaling [101]. Consistent with these mechanistic observations, pan-cancer multi-omics work has further positioned DCAF7 as a poor-prognosis factor and functional driver in hepatocellular carcinoma, where it enhances Wnt/β-catenin output (via inhibitory phosphorylation of GSK-3β) and promotes proliferation/migration [102].

A complementary route to sustaining proliferation is to secure the biosynthetic capacity required for rapid biomass accumulation, and WDR43 exemplifies this “infrastructure” logic. Canonically, WDR43 (Utp5) is an essential nucleolar component of the SSU processome that coordinates early pre-rRNA processing steps required for 18S rRNA production and small ribosomal subunit biogenesis—processes that become rate-limiting when tumors escalate translational demand [51, 103]. Beyond this nucleolar role, an unexpected chromatin-facing function positions WDR43 as a direct regulator of mitogenic transcription: in embryonic stem cells, WDR43 behaves as a chromatin-associated RNA-binding protein that binds promoter-/enhancer-associated nascent RNAs, occupies thousands of active regulatory regions, and interfaces with the Pol II machinery to facilitate pause release and productive elongation. Notably, acute WDR43 depletion rapidly reduces Pol II activity, supporting a proximal, non-secondary role in transcriptional output [40].

### Checkpoint and tumor-suppressor bypass via ubiquitin–proteostasis wiring

Evading growth suppressors frequently involves disabling p53-mediated arrest/apoptosis and rewiring checkpoint enforcement. DDB1, as the adaptor component of the CUL4 E3 ubiquitin ligase platform, controls diverse substrates and has been repeatedly linked to cellular p53 homeostasis. Genetic perturbation of DDB1 activates p53 pathway outputs, consistent with DDB1-containing CRL4 complexes functioning upstream of p53 stability and checkpoint execution [104, 105]. Importantly, tumor-context regulatory layers can further tune this axis: O-GlcNAcylation of DDB1 has been reported to influence p53 stability through proteasomal degradation mechanisms in colorectal cancer cells, providing a biochemical route for suppressor evasion without direct TP53 mutation [106].

FBXW7 is the archetypal WDR substrate receptor in SCF ubiquitin ligases, and its loss promotes growth suppressor evasion by stabilizing multiple oncogenic drivers (e.g., MYC, cyclin E, NOTCH, mTOR pathway components) [107]. Notably, cancer cells can diminish FBXW7 function not only via mutation but also via post-translational destabilization (e.g., E3 ligase-mediated degradation of FBW7 itself), which can phenocopy mutational inactivation and relax checkpoint constraints [108]. Together, DDB1- and FBXW7-centered ubiquitin systems illustrate how WDR proteins shape the “growth suppressor landscape” by controlling the abundance of p53 pathway regulators and proliferative oncoproteins.

### Stress tolerance and death-pathway buffering

Resisting programmed cell death is a central cancer hallmark that enables malignant cells to survive under intrinsic oncogenic stress and extrinsic therapeutic pressures [109]. Tumor cells accomplish this through multiple mechanisms, including the enhancement of pro‑survival signaling, suppression of apoptotic checkpoints, alteration of DNA damage responses, and augmentation of autophagy [110]. WD40 repeat (WDR) proteins, given their structural role as scaffolds and regulators of multi‑protein complexes, have emerged as critical facilitators of these survival mechanisms by stabilizing signaling assemblies, attenuating death signaling pathways, and sustaining cellular stress tolerance [38].

WDR26, a WD40 domain scaffold protein, enhances the efficiency and output of the PI3K/AKT survival pathway, a key anti‑apoptotic axis in cancer. Functional studies demonstrate that WDR26 overexpression promotes AKT activation, which in turn suppresses apoptosis and enhances tumor cell survival, proliferation, and resistance to stress. Conversely, WDR26 knockdown reduces AKT phosphorylation and sensitizes cells to apoptotic triggers, underscoring its role in augmenting survival signaling that counteracts cell death programs [111].

WDR45B (WIPI3) promotes cell-death resistance mainly by safeguarding productive autophagic flux under metabolic and genotoxic stress, thereby limiting proteotoxic and organelle damage that would otherwise amplify mitochondrial apoptosis [112]. WIPI3 (together with WIPI4) can couple nutrient/energy inputs to autophagy initiation through LKB1–AMPK and mTOR signaling, helping sustain early autophagic responses in stressed cells [113]. Mechanistically, WDR45B also operates at a key maturation checkpoint: WDR45/45B are largely dispensable for autophagosome closure but are required for efficient autolysosome formation, in part by binding EPG5 and promoting autophagosome–lysosome fusion [112, 114]. Genetic dissection of the WIPI family further indicates partial redundancy between WIPI3 and WIPI4, which may help preserve autophagy-dependent survival when one branch is compromised [91].

WDR76 has been increasingly linked to cell-death control through its coupling to chromatin and the DNA damage response, positioning it to influence whether genotoxic stress is resolved or converted into apoptotic execution. In mechanistic cell-based studies, WDR76 co-localizes with heterochromatin-associated proteins (including HP1 family members) and is rapidly recruited to laser-induced DNA damage tracks, supporting a direct, proximal role in the cellular damage-response choreography rather than a purely downstream consequence of altered proliferation [115]. Consistent with this positioning, a dedicated disease-focused synthesis highlights WDR76 as a multifunctional WD40 protein potentially involved in DNA damage repair, apoptosis, and cell-cycle regulation, reflecting the complexity of its interaction network and context-dependent outputs [116]. Importantly, recent cancer work suggests that WDR76 can act as a determinant of therapy-induced death in colon cancer: WDR76 was reported to regulate 5-fluorouracil responsiveness via HRAS, with functional data indicating that manipulating WDR76 levels alters drug sensitivity and apoptotic/colony-formation phenotypes in resistant cells, implying that WDR76 loss or attenuation may represent one route by which tumor cells buffer genotoxic stress and acquire cell-death resistance during chemotherapy [117].

### Telomere and senescence programs enabling limitless replicative potential

Replicative immortality is achieved when tumor cells bypass senescence/crisis and maintain telomeres across divisions, most commonly by reactivating telomerase and, in a subset of tumors, by engaging alternative lengthening of telomeres (ALT) [118, 119]. In practice, this hallmark is sustained not only by direct telomere-elongation machinery but also by epigenetic programs that silence senescence checkpoints (e.g., CDKN2A/p16) and by transcriptional networks that couple self-renewal cues to telomerase gene expression [120, 121]. WDR proteins intersect each of these layers through their characteristic roles as β-propeller adaptors in transcriptional complexes, telomerase-trafficking modules, and repressive chromatin machinery.

TBL1X and TBL1XR1 are WD40-containing adaptors that facilitate dynamic exchange of corepressor/coactivator assemblies on chromatin and promote productive Wnt transcription by recruiting β-catenin to Wnt target promoters [122]. This positioning becomes directly relevant to immortality because β-catenin has been shown to directly activate TERT transcription via promoter engagement in stem and cancer contexts, providing a mechanistic bridge between Wnt-driven self-renewal programs and telomerase reactivation [123, 124]. In parallel, telomerase itself can feed into Wnt transcriptional complexes as a cofactor, reinforcing a Wnt–TERT circuit that supports sustained self-renewal-like transcription and long-term proliferative capacity [119]. Together, these data support a model in which TBL1X/TBL1XR1 act as chromatin adaptors that increase the efficiency and persistence of Wnt/β-catenin transcriptional output, thereby facilitating telomerase-linked escape from senescence in tumors that rely on Wnt programs [122, 125].

A second, more direct WDR entry point into immortality is telomerase biogenesis and trafficking. WRAP53β (also known as TCAB1/WDR79) is a WD40 protein essential for recruiting telomerase to Cajal bodies and enabling efficient telomere elongation in human cancer cells, establishing a concrete mechanistic link between a WD40 scaffold and telomerase-dependent immortality [126, 127]. Notably, very recent synthesis work has emphasized TCAB1 as a promoter of tumor growth through telomere maintenance, underscoring continuing interest in this axis as both a biomarker and a vulnerability [128].

Beyond telomere elongation, replicative immortality requires durable suppression of senescence gatekeepers, and EED provides a WDR-dependent epigenetic mechanism to achieve this. As the WD40 “reader” subunit of PRC2, EED recognizes pre-existing H3K27me3 through an aromatic cage and allosterically stimulates EZH1/2 catalytic activity, creating a positive-feedback loop that promotes H3K27me3 propagation and maintenance across Polycomb target loci [129, 130]. This reader–writer coupling supports persistent silencing of pro-senescence programs, including pathways converging on CDKN2A/p16, thereby lowering the probability that oncogenic and replication stress will trigger irreversible arrest. Clinically and translationally, recent work in SMARCB1-deficient sarcomas demonstrates that bypassing EZH2-active-site resistance with allosteric EED inhibition (MAK683) can restore PRC2 dependence and re-engage growth control; notably, loss of CDKN2A correlates with failure to induce p16 after EZH2 inhibition despite on-target PRC2 suppression, highlighting senescence circuitry as a key determinant of long-term proliferative escape [65, 121].

### Hypoxia–HIF axis and vascular remodeling

Angiogenesis is a prerequisite for sustained tumor expansion and dissemination, largely driven by hypoxia-responsive transcriptional programs centered on HIF-1/2 and their downstream effectors such as VEGF family ligands, which collectively remodel endothelial behavior, vascular permeability, and extracellular matrix [131, 132]. While canonical oxygen-sensing (PHD–VHL) governs HIF stability, tumor angiogenesis is equally shaped by “non-canonical” regulators that tune HIF turnover, transcriptional potency, and chromatin accessibility; WDR proteins, as β-propeller scaffolds and chromatin adaptors, recurrently occupy these regulatory layers [133, 134].

Beyond its established roles in sustaining mitogenic transcriptional states and supporting metastatic competence, WDR5 can also promote the angiogenic switch by amplifying the hypoxia-responsive HIF transcriptional program that drives VEGF-family expression. HIF-1α is a central regulator of tumor angiogenesis, activating pro-vascular genes such as VEGFA under intratumoral hypoxia [135]. Mechanistically, a clear WDR5–HIF connection has been demonstrated in cholangiocarcinoma: WDR5 increases overall HIF-1α accumulation through two complementary routes, including a c-Myc–dependent component that promotes HIF1A transcription and an additional Myc-independent component that stabilizes HIF-1α protein, thereby strengthening downstream hypoxia signaling that is inherently pro-angiogenic [136]. This framework is consistent with the broader concept emerging from recent HIF-centric syntheses: non-canonical regulators that enhance HIF abundance or transcriptional competence can materially increase angiogenic output even without altering the canonical PHD–VHL oxygen-sensing axis [137]. In this light, WDR5 may act as an epigenetic amplifier that supports a transcription-permissive chromatin state and enhances the output of hypoxia/HIF-associated vascular effectors, contributing to tumor angiogenic adaptation [138].

In addition to its roles in pathway-responsive transcription and metastatic programs discussed above, TBL1XR1 has a well-defined pro-vascular function that is particularly evident in lymphangiogenesis. In esophageal squamous cell carcinoma, TBL1XR1 overexpression promotes lymphatic endothelial cell migration and tube formation, increases lymphatic vessel formation in vivo, and enhances lymphatic metastasis, whereas TBL1XR1 silencing produces the opposite phenotype. Mechanistically, TBL1XR1 directly induces VEGF-C by binding the VEGF-C promoter, establishing a transcriptional basis for its lymphangiogenic output; functional dissection further indicates that VEGF-C is required for TBL1XR1-driven activation of downstream signaling outputs such as AKT and ERK and for the lymphangiogenesis phenotype itself [139]. Importantly, recent syntheses emphasize angiogenesis/lymphangiogenesis as a recurrent component of TBL1XR1 oncogenic activity across tumor types, positioning the TBL1XR1–VEGF axis as a clinically relevant vulnerability rather than a context-specific observation [139–141].

RACK1 regulates tumor angiogenic competence by directly modulating HIF-1α protein stability and, consequently, the amplitude and persistence of hypoxia-inducible vascular effector expression (including VEGF-axis genes) [142]. The core mechanism is well established: RACK1 binds the PAS-A region of HIF-1α and competes with HSP90, shifting HIF-1α away from chaperone-mediated stabilization and toward assembly with Elongin-C/Elongin-B ubiquitin-ligase components, enabling proteasomal degradation through an oxygen-sensing–independent route; this provides a non-canonical, tunable layer for controlling hypoxia output beyond the PHD–VHL axis [143]. A recent 2025 study further refined this axis by showing that PARylation-associated phosphorylation enhances RACK1 dimerization and, in turn, accelerates ubiquitination-dependent HIF-1α turnover [144]. In line with these findings, a 2025 integrative synthesis proposed that PTM-regulated changes in RACK1 assembly, including Ser146-linked dimerization, govern engagement of Elongin-C–containing ubiquitin machinery and thereby set the steady-state level of HIF-1α, offering a mechanistic basis for dynamic tuning of hypoxia-driven angiogenic output without altering canonical oxygen sensing [57].

### Motility, EMT plasticity, and metastatic colonization

Metastatic progression requires sustained motility, dynamic adhesion turnover, extracellular-matrix engagement, and transcriptional plasticity (often via EMT), enabling tumor cells to invade locally, enter circulation, and colonize distant sites. A growing body of work indicates that WDR proteins contribute to this hallmark through “execution-layer” mechanisms that directly stabilize motility effectors, reprogram RNA/translation outputs that favor dissemination, and rewire ubiquitin–deubiquitin circuitry that sustains pro-invasive signaling states [145, 146].

WDR54 has emerged as a metastasis-proximal WD40 protein whose oncogenic contribution is tightly linked to motility wiring and dissemination-relevant signaling. In bladder cancer, WDR54 is upregulated in tumors and higher expression associates with more advanced stage and lymph-node metastasis; functionally, gain- and loss-of-function studies show that WDR54 enhances migratory/invasive behavior and metastatic output in vivo. Mechanistically, WDR54 acts as a stabilizing scaffold for MEMO1 (mediator of ErbB2-driven cell motility 1) by preventing its ubiquitination-dependent degradation, thereby sustaining MEMO1 protein abundance as a permissive state for motility [147]. Independent evidence in colorectal cancer is consistent with a broader role for WDR54 in aggressive traits: WDR54 is elevated and prognostically relevant, and knockdown suppresses CRC aggressiveness and tumor growth while converging on AKT/ERK-related signaling outputs [148]. A separate biochemical study provides an additional mechanistic layer by showing that transglutaminase 2 can cross-link WDR54 and enhance EGFR pathway activation, offering a plausible route by which WDR54 might reinforce invasion-associated signaling in epithelial tumors [149].

DCAF13 (DDB1- and CUL4-associated factor 13) functions as a CRL4 substrate receptor and has been linked to metastatic competence through pathways that directly sustain cell migration and invasion. In ovarian cancer, DCAF13 is prominently expressed and associated with poor prognosis, and CRISPR/Cas9-mediated DCAF13 deletion suppresses migration; mechanistically, CRL4–DCAF13 targets FRAS1 for polyubiquitination and proteasomal degradation, and FRAS1-dependent engagement of FAK signaling is implicated as a downstream route by which DCAF13 maintains motility-related phenotypes [150]. Evidence that DCAF13 can also support dissemination-relevant behaviors in additional epithelial contexts comes from non–small cell lung cancer, where DCAF13 knockout reduces migration alongside other malignant traits, consistent with a broader role in maintaining an invasion-permissive cellular state [151]. These observations align with recent CRL4-focused syntheses that highlight DCAF-dependent substrate selection as a key determinant of cancer-associated behaviors, including motility programs and pathway rewiring, thereby providing a mechanistic framework for considering DCAF13 as a metastasis-linked CRL4 node rather than a lineage-restricted marker [64].

WDR48 (UAF1) supports invasion and metastasis mainly by enabling deubiquitinase-driven maintenance of EMT signaling. In triple-negative breast cancer, the USP1–WDR48 complex has been shown to potentiate TGF-β–induced EMT and migration; suppression of USP1 or WDR48 reduces Smad2/3 and MAPK pathway phosphorylation and increases TAK1 polyubiquitination, consistent with a model in which USP1–WDR48 stabilizes TAK1 to sustain pro-migratory TGF-β signaling [152]. Mechanistic insight at the protein-complex level has been strengthened by recent work demonstrating that UAF1 binding enhances USP1 activity by stabilizing USP1 and improving access to substrates, providing a concrete biochemical basis for why WDR48 can act as a “licensing” cofactor for metastasis-relevant signaling maintenance [152]. In hepatocellular carcinoma, WDR48 has also been reported to promote migration, invasion, EMT marker changes, and metastatic phenotypes, with evidence implicating a functional requirement for c-MYC in WDR48-driven aggressive behavior [153]. Consistently, a synthesis of USP1 biology highlights the USP1–WDR48 axis as a recurring determinant of migration and invasion across tumor settings, reinforcing the relevance of this WD40–DUB module to metastatic competence [154].

## WDR proteins in specific cancer types

**Fig. 5:**
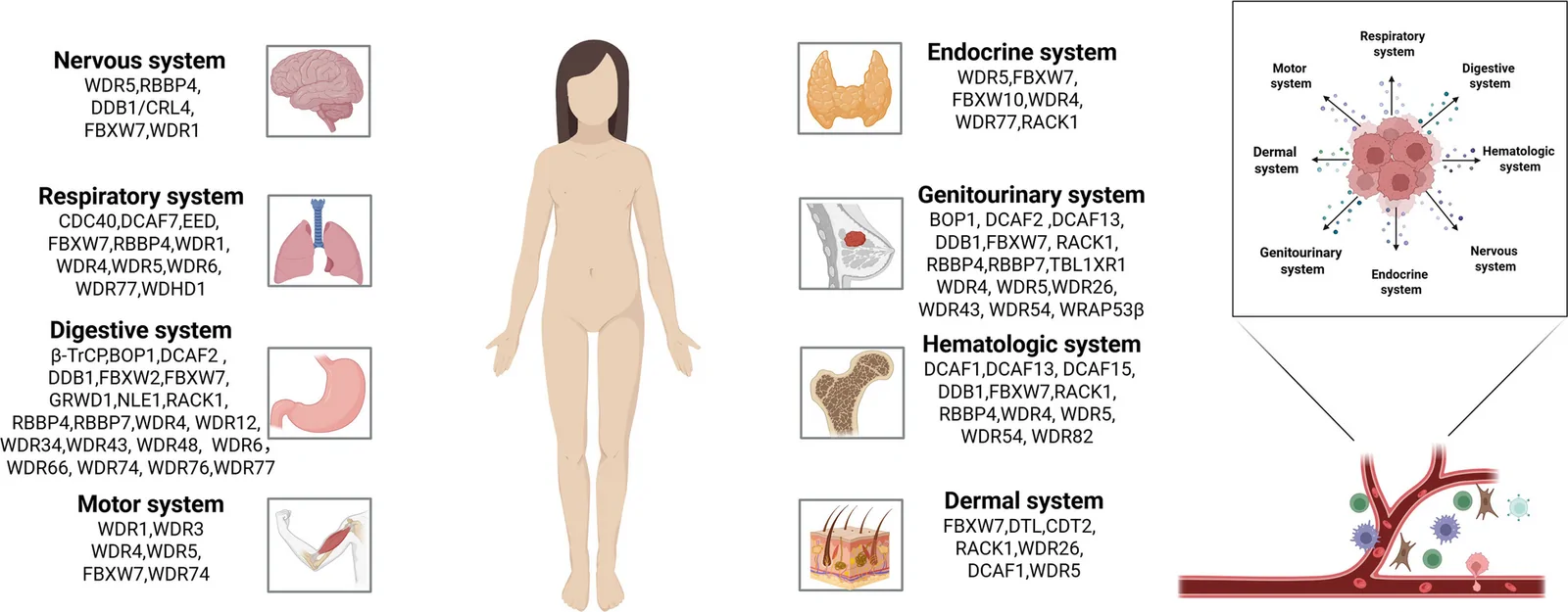
Distribution of cancer-associated WDR proteins across different organ systems. This schematic summarizes the organ system-specific distribution of WD40-repeat (WDR) proteins implicated in human cancers. Eight major anatomical systems are shown, including the respiratory, digestive, hematologic, nervous, endocrine, genitourinary, dermal, and motor systems

With the rapid advances in research on the WDR protein family in recent years, accumulating evidence indicates that WDR proteins play indispensable regulatory roles in tumor initiation, progression, and malignant evolution. In this review, we will systematically categorize cancers by tumor type and delineate the context-dependent functions and potential mechanisms of WDR proteins across distinct oncogenic settings, with the goal of informing future directions for precision diagnosis and targeted therapy (Fig. 5).

### Respiratory system tumors

#### Nasopharyngeal carcinoma

Despite advances in imaging, intensity-modulated radiotherapy, and systemic therapy, nasopharyngeal carcinoma (NPC) still faces unresolved challenges in early detection, biological stratification, recurrence management, and treatment resistance. Plasma EBV DNA is useful for screening and surveillance, but broader clinical implementation still requires standardized algorithms, thresholds, and follow-up strategies [155, 156]. TNM staging does not fully capture biological heterogeneity [157], and recurrent disease often requires high-risk salvage treatment such as re-irradiation [158]. In recurrent or metastatic NPC, PD-1 blockade combined with gemcitabine and cisplatin has improved outcomes, but response heterogeneity and acquired resistance remain major limitations [159, 160]. Against this background, WDR proteins may provide mechanistic information that complements existing clinical markers by linking chromatin regulation, ubiquitin-dependent proteostasis, RNA modification, and stress-adaptive signaling to NPC progression and therapeutic response (Table 1).

**Table 1:** WDR proteins in respiratory system tumors: mechanistic functions and clinical significance

Cancer type	WDR protein	Role	Representative mechanism/pathway	Clinical/translational relevance	Ref
Nasopharyngeal carcinoma	DCAF7	Oncogenic	Promotes USP10-G3BP1 interaction, stabilizes G3BP1 via deubiquitination, enhances stress-granule-like assembly	Chemoresistance, metastasis, poor prognosis	[161]
Nasopharyngeal carcinoma	WDR4	Oncogenic	METTL1-WDR4-mediated m^7^G tRNA modification activates WNT/β-catenin and EMT	Cisplatin/docetaxel resistance, metastasis	[46]
Nasopharyngeal carcinoma	FBXW7	Tumor suppressive	FBP1-FBXW7-mTOR axis suppresses glycolysis	Radiosensitivity/radiotherapy response	[162]
Nasopharyngeal carcinoma	WDHD1	Oncogenic	Associated with cell-cycle/apoptosis control; may regulate ITGAV-related programs	Diagnostic/prognostic candidate	[163]
Nasopharyngeal carcinoma	WDR5	Oncogenic	Chromatin adaptor proposed as miRNA-linked target	Potential therapeutic target	[164, 165]
Lung cancer	WDR5	Oncogenic	Supports oncogenic transcriptional output by elevating SOX9	Tumor growth	[166]
Lung cancer	RBBP4	Oncogenic/context-dependent	Chromatin maintenance; suppression triggers autophagic cell death and alters drug response	Diagnostic/prognostic marker; survival dependency	[167]
Lung cancer	EED	Oncogenic	Allosteric EED inhibition remodels tumor immune microenvironment and suppresses progression	Epigenetic therapeutic target	[168]
Lung cancer	WDR4	Oncogenic	METTL1-WDR4 installs m^7^G tRNA modifications to bias translation toward growth/invasion	Proliferation, invasion, tumorigenicity	[45]
Lung cancer	FBXW7	Tumor suppressive	Loss promotes progression and gefitinib resistance; partial rescue via mTOR co-targeting	Gefitinib resistance	[169]
Lung cancer	WDR1	Oncogenic	Coordinates ADF/cofilin-mediated actin remodeling with YAP activation/nuclear localization	Growth, migration, mechanotransduction	[49]
Lung cancer	WDR77	Oncogenic	PRMT5-WDR77 axis drives invasion-related transcription downstream of TGF-β	Invasion	[170]
Lung cancer	CDC40	Oncogenic/dependency	Spliceosome-related factor required for splicing integrity and survival	Cell survival vulnerability	[171]
Lung cancer	WDR6	Biomarker-like	Correlates with immune infiltration features	Prognostic/immune-context marker	[172]

Rather than acting as a uniform class of biomarkers, WDR proteins appear to contribute to NPC through distinct mechanistic identities. DCAF7 functions as a scaffold-like regulator of cisplatin resistance by promoting the USP10-G3BP1 interaction, stabilizing G3BP1 through deubiquitination, and supporting stress-granule-associated chemoresistance and metastasis [161]. METTL1 and its WD40 partner WDR4 represent an RNA-modification module, in which m7G tRNA modification promotes WNT and β-catenin signaling, EMT, and resistance to cisplatin and docetaxel [46]. FBXW7, as a WD40-containing F-box substrate receptor, influences radiosensitivity through the FBP1-FBXW7-mTOR axis and metabolic control [162]. Other WDR proteins, including WDHD1 and WDR5, are more closely linked to proliferative or transcriptional states, suggesting potential value in mechanism-informed stratification rather than as stand-alone clinical markers [163–165]. Collectively, these studies suggest that WDR proteins may refine NPC classification and expose resistance vulnerabilities, but their clinical relevance will depend on validation in defined patient subgroups and integration with established indicators such as EBV DNA, TNM stage, and treatment-response profiles.

#### Lung cancer

Lung cancer management has been reshaped by low-dose CT screening, molecular profiling, targeted therapy, and immunotherapy, yet clinical impact remains limited by imperfect risk stratification and frequent treatment resistance. Low-dose CT reduces mortality but is accompanied by false positives, unnecessary procedures, and overdiagnosis [173]. Liquid biopsy is increasingly used for genotyping, minimal residual disease monitoring, and perioperative decision-making, but sensitivity in low-burden disease and cross-platform standardization remain unresolved [174, 175]. In advanced disease, EGFR-targeted therapy is undermined by acquired resistance, including secondary EGFR mutations, MET bypass activation, and histologic transformation, while PD-L1 and TMB incompletely predict immunotherapy response [176–178]. In this context, WDR proteins may represent context-dependent regulatory hubs that help define distinct biological states of lung cancer, including proliferative dependency, metastatic competence, immune responsiveness, and resistance evolution (Table 1).

In lung cancer, current evidence is better interpreted through the lens of tumor-state dependency and adaptive rewiring than through isolated marker discovery. Several WDR proteins participate in chromatin-governed survival programs. WDR5 acts as a scaffold-like chromatin adaptor that sustains oncogenic transcriptional output, including SOX9-associated lung adenocarcinoma progression [166]. RBBP4 and EED further indicate that WD40-containing chromatin regulators may influence tumor cell survival, drug response, and immune-related tumor states, although their clinical utility remains to be defined in molecularly annotated cohorts [167, 168]. Beyond chromatin regulation, METTL1-WDR4 links RNA modification to translational control by installing m7G tRNA modifications that favor proliferative and invasive programs [45], whereas FBXW7 represents a proteostasis-related tumor suppressor whose loss promotes NSCLC progression and gefitinib resistance, partly through mTOR-associated signaling [169]. Other WDR proteins are more closely connected with phenotype execution. WDR1 couples actin remodeling to YAP activation and motility, WDR77 serves as a PRMT5 cofactor in TGFβ-associated invasion programs, CDC40 maintains spliceosome integrity required for cell survival, and WDR6 correlates with prognosis and immune infiltration features [49, 170–172]. These findings suggest that WDR proteins may help define biologically distinct lung cancer states, including chromatin dependency, translational rewiring, proteostasis imbalance, cytoskeleton-YAP-driven motility, splicing vulnerability, and immune-context variation. However, their clinical application will require evidence that these WDR-associated states add actionable information beyond established genomic drivers, PD-L1, TMB, MRD status, and known resistance mechanisms.

### Digestive system tumors

#### Esophageal cancer

Esophageal cancer (EC) remains clinically challenging because early detection, treatment selection, and relapse surveillance are still suboptimal. Endoscopy is central to diagnosis, but population-level screening is limited by access, adherence, and risk stratification, leaving many patients diagnosed at locally advanced stages [179]. Although multimodality therapy has improved disease control, responses to neoadjuvant treatment and relapse risk after curative-intent therapy remain heterogeneous [180]. ctDNA-based minimal residual disease monitoring shows promise for postoperative prognostication, but sensitivity in low-burden disease and standardized decision thresholds remain unresolved [181]. Immunotherapy has expanded treatment options, yet durable benefit is inconsistent and biomarker-guided selection remains incomplete [182]. In this setting, WDR proteins may help define therapy-relevant EC states shaped by translational control, proteostasis imbalance, chromatin-driven invasion, and treatment resistance (Table 2).

**Table 2:** WDR proteins in digestive system tumors: mechanistic functions and clinical significance

Cancer type	WDR protein	Role	Representative mechanism/pathway	Clinical/translational relevance	Ref
Esophageal cancer	WDR4	Oncogenic	METTL1-WDR4-mediated m^7^G tRNA modification rewires translation via RPTOR-ULK1/autophagy axis	Tumorigenesis, stress adaptation	[47]
Esophageal cancer	FBXW7	Tumor suppressive	FBXW7-MAP4-ERK circuit restrains progression	Progression; pathway-matched vulnerability	[183]
Esophageal cancer	RBBP4	Oncogenic	KTN1-AS1 binds RBBP4 and strengthens RBBP4-HDAC1 interaction to repress E-cadherin	EMT/invasion	[184]
Esophageal cancer	RBBP7	Oncogenic	NuRD-related histone-binding subunit promoting migration/invasion	Poor prognosis, invasion	[185]
Esophageal cancer	WDR77	Oncogenic	PRMT5-WDR77 drives squamous carcinoma proliferation throughΔNp63alpha-p21 axis	Proliferation/checkpoint control	[186]
Esophageal cancer	WDR66	Biomarker-like	Associated with ESCC risk stratification	Risk stratification	[187]
Esophageal cancer	RACK1	Oncogenic	EMT-linked WD40 scaffold driving progression	Progression, poor prognosis	[188]
Esophageal cancer	WRAP53β/WDR79	Oncogenic/biomarker	Overexpression correlates with deeper invasion and lymph-node metastasis	Aggressive clinicopathologic features	[189]
Gastric cancer	WDR74	Oncogenic	ATF5-WDR74 axis enhances beta-catenin nuclear accumulation and Wnt transcription	Stemness/aggressive behavior	[190]
Gastric cancer	GRWD1	Oncogenic	Upregulates ADAM17 and strengthens Notch signaling via NICD release	Progression	[191]
Gastric cancer	BOP1	Oncogenic	PeBoW/ribosome biogenesis factor; silencing induces p53/p21-mediated G0/G1 arrest and apoptosis	Proliferation; ribosome-stress vulnerability	[192]
Gastric cancer	FBXW2	Tumor suppressive	Promotes ubiquitination-dependent degradation of WASL	Restrains viability and metastasis	[193]
Gastric cancer	WDR62	Oncogenic/biomarker	Frequently overexpressed and associated with poor differentiation	Poor prognosis marker	[194]
Hepatocellular carcinoma	WDR76	Tumor suppressive	RAS-binding protein promoting RAS degradation	Suppresses malignant phenotypes	[195]
Hepatocellular carcinoma	WDR34	Oncogenic	Enhances Wnt/β-catenin transcriptional activity	Growth and migration	[196]
Hepatocellular carcinoma	WDR48	Oncogenic	Promotes progression by stabilizing c-Myc	Migration, invasion, EMT/metastasis	[153]
Hepatocellular carcinoma	NLE1	Oncogenic/biomarker	Promotes proliferation	Diagnostic and prognostic biomarker	[197]
Hepatocellular carcinoma	β-TrCP	Oncogenic context	WD40 substrate receptor implicated in carcinogenesis through signaling substrate turnover	Conceptual proteostasis framework/drug response	[198]
Pancreatic ductal adenocarcinoma	DTL/CDT2/DCAF2	Oncogenic	CRL4 substrate receptor drives K48-linked ubiquitination and degradation of SMAD4 with Wnt/β-catenin activation	Progression	[199]
Pancreatic ductal adenocarcinoma	DDB1	Oncogenic	Promotes proliferation, EMT and gemcitabine resistance; inverse relation with dCK	Chemoresistance/poor prognosis	[200]
Pancreatic ductal adenocarcinoma	WDR79	Oncogenic	Maintains UHRF1-dependent suppression of SIRT4 to support aerobic glycolysis	Growth and motility under stress	[201]
Pancreatic ductal adenocarcinoma	WDR12	Oncogenic/context	Enriched in nucleolar/pre-ribosomal modules	Ribosome biogenesis dependence	[202]
Pancreatic ductal adenocarcinoma	WDR43	Oncogenic/biomarker	Pan-cancer association with poor outcome and immune features in PDAC	Predictive/prognostic candidate	[203]
Colorectal cancer	WDR43	Oncogenic	Maintains vimentin and EMT-like program	Proliferation, invasion, tumorigenesis; diagnostic/therapeutic candidate	[204]
Colorectal cancer	WDR12	Oncogenic	WDR12-RAC1 axis favors proliferative and anti-apoptotic outputs	Progression	[205]
Colorectal cancer	TBL1XR1	Oncogenic	Promotes EMT through β-catenin	Postoperative recurrence, poor DFS	[206]
Colorectal cancer	RBBP4	Oncogenic	Drives malignant progression by increasing Wnt/β-catenin activity	Progression	[206]
Colorectal cancer	RBBP7	Tumor suppressive/context-dependent	lncRNA FIT recruits RBBP7 to enhance p53 acetylation and FAS transcription	Apoptosis regulation	[207]
Colorectal cancer	WDR62	Oncogenic	Promotes oxaliplatin resistance and DNA damage repair through MAPK activation	Oxaliplatin resistance	[208]
Colorectal cancer	FBXW7	Tumor suppressive	Alterations contribute to resistance to targeted therapies	Therapy resistance	[208]
Colorectal cancer	RACK1	Oncogenic	Supports proliferative and invasive behavior	Progression	[209]
Colorectal cancer	WRAP53	Biomarker-like	Associated with prognosis and radiotherapy response in rectal cancer	Stratification/RT response	[210]

Current evidence for WDR proteins in EC is still dominated by ESCC studies, but several findings already suggest biologically meaningful points of convergence. One relatively consistent theme is that ESCC cells may exploit WDR-associated RNA and protein homeostasis programs to sustain growth under stress. The METTL1-WDR4 complex promotes m^7^G tRNA modification and alters translational output, with one study linking this process to the RPTOR-ULK1 autophagy axis [47]. In parallel, loss of the WD40-containing F-box protein FBXW7 increases MAP4 abundance and ERK phosphorylation, defining a FBXW7-MAP4-ERK circuit that supports ESCC progression and may expose downstream vulnerabilities in FBXW7-defective tumors [183]. A second theme involves chromatin control of invasion. SOX2-induced KTN1-AS1 recruits RBBP4 and enhances its interaction with HDAC1, resulting in E-cadherin repression and EMT activation [184]. RBBP7, another NuRD-associated WD40 subunit, is also linked to poor prognosis and increased migration and invasion, suggesting that WD40-containing chromatin complexes may help maintain invasive transcriptional states in ESCC [185]. WDR77 adds a related but distinct layer by acting as the WD40 cofactor of PRMT5 in the ΔNp63α-p21 axis, connecting arginine methylation to squamous cell proliferation [186]. Other reported candidates, including WDR66, RACK1, and WRAP53β, broaden the clinical relevance of WD40 biology to risk stratification, EMT-associated progression, invasion depth, and lymph-node metastasis, but these observations remain less mature mechanistically and should be interpreted as hypothesis-generating rather than clinically established [187–189]. Overall, current studies support a mechanism-focused interpretation of WDR proteins in EC, with the strongest evidence involving translational rewiring, FBXW7-dependent proteostasis, chromatin-mediated EMT, and PRMT5-WDR77-linked cell-cycle regulation. Whether these mechanisms can improve prognostic assessment or guide treatment selection beyond existing clinical and molecular tools remains to be established.

#### Gastric cancer

Gastric cancer (GC) remains clinically challenging because many patients are diagnosed at advanced stages, and early risk stratification is still insufficiently precise. Endoscopy is the main tool for screening and surveillance in high-risk populations, but its effectiveness depends on appropriate population selection and standardized follow-up strategies [211, 212]. Circulating tumor DNA is being explored for perioperative risk assessment and minimal residual disease monitoring, although low tumor burden, platform variability, and undefined clinical thresholds continue to limit routine use [213]. Biomarker-guided therapy has expanded treatment options, but actionable subgroups remain fragmented, and durable responses are often limited by tumor heterogeneity, adaptive resistance, and microenvironmental remodeling [214]. Within this setting, WD40 repeat proteins may be useful for understanding how chromatin regulation, ubiquitin-dependent proteostasis, and other regulatory programs shape GC progression, therapeutic vulnerability, and resistance patterns (Table 2).

In GC, available studies connect WDR proteins mainly with developmental signaling, ribosome biogenesis, and ubiquitin-dependent proteostasis. WDR74 has been linked to an ATF5-driven program that promotes β-catenin nuclear accumulation and strengthens Wnt transcriptional activity, thereby supporting stemness-associated aggressive behavior [190]. GRWD1 provides another example of WDR involvement in developmental pathway activation, as it upregulates ADAM17, increases NICD release, and enhances Notch signaling during GC progression [191]. Growth control is also influenced by BOP1, a WD40-containing component of the PeBoW ribosome-biogenesis complex. BOP1 depletion induces G0/G1 arrest and apoptosis together with p53 and p21 upregulation, suggesting that ribosome-biogenesis stress may expose a proliferative vulnerability in GC cells [192]. In contrast, FBXW2 acts through substrate regulation: this WD40-type F-box receptor interacts with WASL and promotes its ubiquitination-dependent proteasomal degradation, thereby restraining malignant viability and metastatic potential in experimental models [193]. WDR62 has been reported to be frequently overexpressed in GC and associated with poor differentiation and unfavorable prognosis, although its mechanistic role remains less well defined [194].Collectively, these findings suggest that WDR proteins participate in several GC-relevant regulatory programs, particularly Wnt and Notch signaling, ribosome-biogenesis control, and SCF-linked substrate turnover. Their clinical relevance will be clearer when these mechanisms are tested in well-annotated cohorts and compared with established pathological and molecular risk factors.

#### Hepatocellular carcinoma

Hepatocellular carcinoma (HCC) remains difficult to manage because early detection, recurrence monitoring, and treatment selection are still imperfect. Current surveillance largely depends on ultrasound with or without AFP every six months, but small lesions may be missed, particularly when visualization is suboptimal. Abbreviated MRI and composite biomarker scores are being explored, although access, standardization, and implementation remain uneven [215, 216]. Recurrence after resection or ablation is common, and ctDNA-based minimal residual disease monitoring may provide earlier relapse information, but sensitivity in low-burden disease, assay variability, and undefined clinical thresholds still limit routine use [217]. In advanced HCC, immunotherapy-based combinations have improved systemic therapy, yet responses remain heterogeneous and resistance is frequent, partly reflecting tumor and microenvironmental diversity and the lack of reliable predictive biomarkers [218, 219]. These challenges highlight the need to better define the mechanisms underlying HCC recurrence, therapeutic resistance, and biological heterogeneity. WDR proteins are therefore relevant candidates for investigation because they may participate in proteostasis control, RAS and Wnt/β-catenin signaling, Myc stability, and stress-adaptive programs linked to HCC progression. (Table 2).

Available HCC studies suggest that WDR proteins influence tumor progression through several distinct regulatory routes. WDR76 acts as a cytoplasmic RAS-binding factor that promotes RAS degradation, thereby restraining oncogenic signaling through protein stability control rather than transcriptional regulation alone [195]. WDR34 shows an opposite pattern, enhancing Wnt and β-catenin transcriptional activity and promoting HCC cell growth and migration, which links this WDR protein to invasive and recurrence-prone phenotypes [196]. WDR48 adds another layer of proteostasis control by stabilizing c-Myc, consistent with its role in deubiquitinase-associated regulation of oncogenic protein output [153]. NLE1 has been proposed as an independent diagnostic and prognostic marker and has been shown to promote HCC proliferation, although its mechanistic position remains less clearly defined than those of WDR76, WDR34, or WDR48 [197]. β-TrCP proteins represent the clearest substrate-receptor model in this section, as WD40-containing SCF E3 ligase components that regulate key signaling substrates during carcinogenesis [198]. Overall, these findings suggest that HCC-associated WDR proteins should be interpreted according to their positions in RAS proteostasis, Wnt/β-catenin signaling, Myc stabilization, and SCF-mediated substrate control. Their clinical relevance will require validation in well-defined HCC cohorts and comparison with established variables such as tumor stage, liver function, AFP, molecular subtype, ctDNA status, and treatment response.

#### Pancreatic ductal adenocarcinoma

Pancreatic ductal adenocarcinoma (PDAC) is characterized by a narrow clinical window, as early lesions are usually silent and many patients are diagnosed only after local invasion or metastasis. CA19-9 is widely used but lacks sufficient accuracy for early detection or stand-alone surveillance, while imaging-based screening in high-risk groups remains resource intensive and may miss very small or indeterminate lesions [220, 221]. ctDNA-based assays are being explored for perioperative risk assessment and minimal residual disease detection, but low tumor burden, platform heterogeneity, and non-standardized thresholds limit routine use [222, 223]. Therapeutically, chemotherapy remains the backbone but provides limited survival benefit in metastatic disease, and immunotherapy has shown only modest activity in most patients because of the dense, immunosuppressive PDAC microenvironment [224, 225]. These features support further investigation of WDR proteins in PDAC, particularly in relation to protein turnover, EMT-associated drug response, metabolic adaptation, and ribosome-biogenesis demand (Table 2).

The available WDR literature mirrors several defining features of PDAC biology. DTL/CDT2 provides a direct example of ubiquitin-mediated pathway rewiring. As a WD40 substrate receptor in CRL4 ubiquitin ligase complexes, DTL promotes K48-linked ubiquitination and proteasomal degradation of SMAD4, thereby facilitating Wnt/β-catenin activation and malignant progression [199]. DDB1 reflects a broader CRL4 adaptor function. Its high expression is associated with poor prognosis and experimentally linked to enhanced proliferation, reduced gemcitabine-induced apoptosis, EMT activation, and lower dCK expression, placing it at the intersection of proteostasis, epithelial plasticity, and gemcitabine response [200]. WDR79 points to a metabolic layer of regulation by maintaining UHRF1-dependent repression of SIRT4 and sustaining glycolytic effectors, thereby supporting PDAC growth and motility under stress [201]. Proteomic studies further suggest that pancreatic cancer cells rely on nucleolar and pre-ribosomal programs involving WD40 proteins such as WDR12 and WDR43, consistent with the high biosynthetic and translational demand of this tumor type [202]. In addition, pan-cancer analyses associate elevated WDR43 with poorer outcome and immune-related features in pancreatic adenocarcinoma, although this remains more correlative than mechanistically resolved in PDAC [203]. Taken together, the strongest PDAC-related signals place WDR proteins in CRL4-dependent substrate control, SMAD4/Wnt signaling, gemcitabine-associated EMT programs, glycolytic adaptation, and ribosome biogenesis. At this stage, these findings are best used to guide pathway-based prioritization and combination hypotheses, while their value for clinical stratification will need confirmation alongside CA19-9, imaging, ctDNA, molecular subtype, and treatment-response data.

#### Colorectal cancer

Colorectal cancer (CRC) is one of the most preventable malignancies, yet screening and post-treatment management still face important limitations. Population screening must balance access, false positives, and colonoscopy burden [226]. After curative-intent surgery, ctDNA has become a powerful indicator of molecular residual disease and recurrence risk, but assay standardization, actionable thresholds, and integration into adjuvant treatment decisions remain unresolved [227]. In metastatic CRC, therapeutic pressure rapidly selects resistant clones, and most microsatellite-stable tumors respond poorly to immune checkpoint blockade [228]. CRC therefore provides a clinically relevant setting in which WDR proteins can be examined as contributors to relapse-prone and therapy-resistant tumor states, particularly through their roles in chromatin regulation, ubiquitin-dependent proteostasis, nucleolar growth, and stress adaptation (Table 2).

In CRC, WDR proteins are best understood as regulators of several tumor-state programs rather than as isolated prognostic candidates. WDR43 supports an EMT-like phenotype by maintaining vimentin expression, and its depletion suppresses proliferation, migration, invasion, tumorigenesis, and increases apoptosis, placing WDR43 close to invasive plasticity control [204]. WDR12 provides a related nucleolar-growth axis, where a WDR12-RAC1 relationship promotes proliferative and anti-apoptotic outputs, linking ribosome-associated WD40 biology with small-GTPase survival signaling [205]. Wnt-dependent transcriptional competence is another recurring theme. TBL1XR1 is associated with postoperative recurrence and poor disease-free survival and can promote β-catenin-dependent EMT, while RBBP4 enhances Wnt/β-catenin pathway activity, suggesting that WD40 chromatin adaptors help maintain Wnt-permissive tumor states in CRC [206]. By contrast, RBBP7 is involved in apoptotic checkpoint regulation: the lncRNA FIT recruits RBBP7 to enhance p53 acetylation and FAS transcription, showing how a WD40 histone-binding subunit can tune p53-dependent apoptosis [207]. Drug-resistance biology is represented by WDR62, which promotes oxaliplatin resistance and DNA damage repair through MAPK activation, and by FBXW7, a WD40-containing F-box substrate receptor whose alterations can contribute to resistance to targeted therapy [208]. Additional WD40 proteins broaden this landscape, including RACK1, a scaffold that supports CRC proliferation and invasion, and WRAP53, which has been linked to prognosis and radiotherapy response in rectal cancer [209, 210]. These findings position WDR proteins within several CRC-relevant biological programs, including EMT plasticity, Wnt-permissive chromatin regulation, nucleolar growth control, p53-mediated apoptosis, and therapy-resistance networks. Their potential contribution to CRC management is therefore likely to lie in refining existing molecular and clinical frameworks, rather than replacing established variables such as TNM stage, ctDNA status, MSI/MMR status, RAS/BRAF alterations, and treatment-response assessment.

### Urogenital system tumors

#### Breast cancer

Breast cancer (BC) care is increasingly biomarker-driven, but important gaps remain in detection, monitoring, and durable disease control. Screening reduces mortality, yet its performance varies with baseline risk and breast density, and supplemental imaging for dense breasts must still balance earlier detection against false positives and downstream procedures [229, 230]. Circulating tumor DNA is being explored for perioperative risk assessment and minimal residual disease surveillance, but low disease burden, inter-assay variability, and undefined action thresholds continue to limit routine clinical implementation [231]. At the therapeutic level, endocrine resistance remains a major obstacle in hormone receptor-positive disease, whereas immunotherapy benefit is largely confined to selected subsets and remains difficult to predict precisely [232, 233]. These limitations shift attention from single-marker discovery toward regulatory mechanisms that shape endocrine escape, metastatic competence, stem-like states, and variable drug sensitivity, including those organized by WD40 repeat proteins (Table 3).

**Table 3:** WDR proteins in urogenital system tumors: mechanistic functions and clinical significance

Cancer type	WDR protein	Role	Representative mechanism/pathway	Clinical/translational relevance	Ref
Breast cancer	WDR5	Oncogenic	Epigenetic dependency supporting ribosomal protein gene expression/global translation	Metastasis; combination with mTOR inhibition	[234]
Breast cancer	DCAF13	Oncogenic	Controls PERP proteostasis and K63-linked ubiquitination of RPA194 to stimulate rRNA transcription	Proliferation and migration	[235]
Breast cancer	WDR26	Oncogenic	Scaffold coordinating GPCR-driven PI3K-AKT activation	Growth, migration, invasion	[7]
Breast cancer	RACK1	Oncogenic	Promotes stem-like properties through E2F1-SOX2 axis	Tumorigenesis/stemness	[236]
Breast cancer	FBXW7	Context-dependent/pro-oncogenic in cited axis	SOX4-driven FBXW7 upregulation promotes GATA3 turnover and reinforces c-Myc programs	Tamoxifen resistance, dormancy, stemness	[237, 238]
Breast cancer	RBBP4	Oncogenic/biomarker	Higher expression associated with LN metastasis and poorer survival	Prognosis	[239]
Breast cancer	RBBP7	Oncogenic	Super-enhancer-driven RBBP7 recruits NuRD components	Metastasis and stem-like traits	[240]
Ovarian cancer	DCAF13	Oncogenic	Enhances FRAS1 ubiquitination and downstream FAK-AKT signaling	Proliferation, migration, relapse	[150]
Ovarian cancer	DDB1	Oncogenic	CUL4 adaptor modulating mitochondrial dynamics and mitophagy	Poor prognosis, chemoresistance	[241, 242]
Ovarian cancer	WDR5	Oncogenic	BAG3-WDR5-dependent activation of GALNT10 in platinum-resistant stem-like state	Drug tolerance/platinum resistance	[243]
Ovarian cancer	RACK1	Oncogenic	Stabilized by low SMURF2 and PCAF-mediated acetylation, sustaining pro-tumor signaling	Growth and stress adaptation	[244]
Ovarian cancer	WRAP53β/TCAB1/WDR79	Context-dependent low expression adverse	Reduced expression associates with attenuated DDR and poorer survival	Prognosis/treatment outcome	[245]
Ovarian cancer	TBL1XR1	Oncogenic/biomarker	Overexpression correlates with VEGF-C and lymph node metastasis	Poor prognosis, metastatic patterning	[140]
Ovarian cancer	FBXW7	Tumor suppressive/context	Recurrently altered WD40 substrate receptor linked to prognosis and therapy resistance	Relapse risk/therapy resistance	[246]
Cervical cancer	WDR5	Oncogenic	Upstream of YAP1-CTGF program; also upregulated by noncoding RNAs	Growth and invasion	[247, 248]
Cervical cancer	RACK1	Oncogenic	Promotes glycolysis-dependent AKT-mTOR signaling and lymph node metastasis; circVPRBP destabilizes RACK1	Lymphangiogenesis/nodal dissemination	[249, 250]
Cervical cancer	TBL1XR1	Oncogenic	Linked to dissemination through lncRNA-regulated circuits	Metastatic propensity	[251]
Cervical cancer	CDT2/DTL	Oncogenic	TRIM22-CDT2-SET8 axis mediates p53/Rb outputs in HPV-positive CC	Growth and survival	[252, 253]
Cervical cancer	FBXW7	Tumor suppressive	Mutations promote proliferation and migration	Progression/possible therapy response	[254]
Cervical cancer	WDR4	Oncogenic/context	METTL1-dependent m^7^G tRNA modification implicated in aggressive translational rewiring	Poor prognosis	[255, 256]
Renal cell carcinoma	BOP1	Oncogenic	Ribosome biogenesis factor linked to aggressive ccRCC and adverse prognosis	Proliferative/stress-adaptive translation	[257]
Renal cell carcinoma	WDR43	Biomarker-like	Associated with outcome and immune contexture	Systems-level marker	[203]
Renal cell carcinoma	DTL/CDT2/DCAF2	Oncogenic	CRL4 receptor supporting replication licensing and S-phase stress tolerance	Malignant progression	[258, 259]
Renal cell carcinoma	FBXW7	Tumor suppressive	Constrains progression through oxidative DNA damage control and AKT wiring	Growth-permissive signaling restraint	[260]
Renal cell carcinoma	RBBP7	Context-dependent	BAP1 engages YY1-RBBP7-associated complex to reshape transcription	Kidney cancer chromatin biology	[261]
Renal cell carcinoma	WDR4	Oncogenic/context	METTL1-WDR4 m^7^G-writing module boosts tRNA functionality and protein synthesis	Translation-linked vulnerability	[262]
Bladder cancer	WDR54	Oncogenic	Stabilizes MEMO1 by limiting ubiquitination	Tumorigenesis, metastasis, cisplatin resistance	[147]
Bladder cancer	WDR5	Oncogenic	OICR-9429 disrupts WDR5-MLL complex, suppresses H3K4 methylation-linked malignant traits and PD-L1-linked immune evasion	Cisplatin responsiveness; immune evasion	[263]
Bladder cancer	WDR4	Oncogenic	Elevated and promotes lymph node metastasis/proliferation via m^7^G-linked translation axis	Aggressive disease	[264]
Bladder cancer	DTL	Oncogenic	Drives malignant progression through AKT-mTOR and cell-cycle programs	Poor prognosis	[265]
Bladder cancer	DDB1	Context-dependent	PRPF19-linked axis contributes to DNA repair and gemcitabine sensitivity patterns	Chemotherapy response	[266]
Bladder cancer	FBXW7	Tumor suppressive	Promotes degradation of ZMYND8	Restrains progression	[267]

In BC, WDR proteins are most consistently linked to epigenetic dependency, ubiquitin-mediated proteostasis, signaling scaffold function, and stemness regulation. WDR5 acts as an epigenetic scaffold in triple-negative models, supporting ribosomal protein gene expression and global translation; its combination with mTOR pathway inhibition suggests a potential vulnerability in tumors dependent on elevated biosynthetic output [234]. DCAF13 represents a CRL4-associated WD40 substrate receptor. It supports proliferation and migration by regulating PERP proteostasis and by promoting K63-linked ubiquitination of the Pol I subunit RPA194, thereby enhancing rRNA transcription and processing [235]. WDR26 illustrates a signaling-scaffold role, assembling GPCR-linked PI3K-AKT activation to promote tumor growth, migration, and invasion [7]. Stem-like phenotypes are also shaped by WD40 scaffolds, as RACK1 promotes tumorigenic and stem-like properties through an E2F1-SOX2 axis [236]. Endocrine therapy resistance intersects with WD40 substrate recognition through FBXW7, whose SOX4-driven upregulation promotes GATA3 turnover, stemness, dormancy, and tamoxifen resistance while reinforcing c-Myc-centered programs [237, 238]. Chromatin-associated WD40 proteins add another layer: RBBP4 expression correlates with lymph-node metastasis and poorer survival, whereas super-enhancer-driven RBBP7 supports metastasis and stem-like traits by increasing NuRD complex recruitment [239, 240]. These findings suggest that WDR proteins contribute to BC progression by supporting subtype-specific programs of epigenetic adaptation, proteostasis control, PI3K-AKT signaling, stem-like plasticity, and endocrine escape. Their clinical value is likely to depend on whether a given WDR-linked circuit can be matched to relapse risk, drug sensitivity, or combination-treatment design.

#### Ovarian cancer

Ovarian cancer (OC) remains a relapse-driven malignancy in which early detection and durable disease control are both difficult to achieve. Broad screening strategies have not provided reliable early diagnosis because of limited test accuracy and false-positive burden [268]. After surgery and platinum-based chemotherapy, recurrence is common; ctDNA-based minimal residual disease monitoring may detect relapse risk earlier, but its routine use is still limited by low-volume disease sensitivity and the absence of standardized cutoffs linked to specific management decisions [269]. Therapeutically, PARP inhibitors have improved outcomes in selected patients, but resistance is frequent and biologically heterogeneous, while immune checkpoint blockade has shown limited activity in most epithelial OCs [270]. These features make OC a useful setting for examining how WD40 repeat proteins participate in relapse-prone chromatin states, ubiquitin-mediated proteostasis, mitochondrial stress adaptation, DNA damage response, and metastatic signaling (Table 3).

Several WDR proteins in OC map onto mechanisms that are closely related to recurrence and treatment tolerance. DCAF13 acts as a CRL4-associated WD40 substrate receptor and promotes proliferation and migration by enhancing FRAS1 ubiquitination, thereby activating FAK-AKT signaling and adhesion-linked growth programs [150]. DDB1 represents the core CUL4 adaptor arm of this ubiquitin-ligase system; its high expression is associated with poor prognosis and chemotherapy resistance, and mechanistic studies link it to mitochondrial dynamics and mitophagy, processes that help tumor cells survive therapeutic stress [241, 242]. In platinum-resistant stem-like states, WDR5 functions as a chromatin scaffold in the BAG3-WDR5-GALNT10 axis, providing a route by which epigenetic regulation can reinforce stemness and drug tolerance [243]. RACK1 illustrates a signaling-scaffold mechanism: reduced SMURF2 and PCAF-mediated acetylation at K130 stabilize RACK1 by limiting ubiquitination, thereby sustaining pro-tumor growth and stress-response signaling in OC [244]. WRAP53β/TCAB1/WDR79 adds a genome-maintenance dimension, as reduced expression is associated with weakened DNA damage response features and poorer survival, consistent with a scaffold role relevant to treatment outcome [245]. TBL1XR1 is better interpreted as a transcriptional co-regulator linked to metastatic patterning, with overexpression associated with poor prognosis, lymph-node metastasis, and higher VEGF-C expression in serous epithelial OC [140]. FBXW7, a WD40-containing F-box substrate receptor, further illustrates how altered substrate turnover may contribute to prognosis and therapy resistance across gynecologic malignancies, including OC [246].Together, these observations suggest that WDR proteins contribute to the biological background in which OC recurrence and drug tolerance emerge, spanning ubiquitin-dependent substrate turnover, stem-like chromatin programs, mitochondrial adaptation, DNA damage response, and adhesion- or VEGF-C-related metastatic signaling. Future studies should clarify which of these WDR-linked processes are clinically informative in defined OC subgroups and whether they can support more effective combination strategies.

#### Cervical cancer

Cervical cancer (CC) prevention has improved with primary high-risk HPV testing, but triage and post-treatment risk assessment remain challenging. Many HPV-positive women have transient infections, and current workflows may still lead to unnecessary colposcopy or overtreatment. Dual-stain p16/Ki-67, extended genotyping, and DNA methylation assays can improve specificity, but scalable triage pathways remain uneven across settings [271, 272]. For high-risk locally advanced disease, chemoradiotherapy remains central, and pembrolizumab has improved outcomes, although relapse still occurs [273]. Tumor-informed circulating HPV ctDNA can detect molecular residual disease after chemoradiation, but limited sensitivity in low-volume disease and non-standardized intervention thresholds restrict routine use [274]. These gaps highlight the need to clarify mechanisms linking HPV-driven transformation with residual disease, metastasis, and treatment tolerance, including WD40 repeat protein-associated regulatory programs (Table 3).

In CC, available studies link WDR proteins to several processes that are closely related to invasion, lymphatic dissemination, and survival of HPV-positive tumor cells. WDR5 acts as a chromatin adaptor that supports a YAP1-CTGF transcriptional program and promotes growth and invasion; noncoding RNA-mediated upregulation of WDR5 further strengthens its association with CC progression [247, 248]. RACK1/GNB2L1 functions as a signaling scaffold in lymphatic spread. It promotes lymph-node metastasis through glycolysis-dependent AKT-mTOR signaling, whereas circRNA-mediated destabilization of RACK1, by blocking O-GlcNAcylation-dependent stabilization, suppresses Galectin-1-associated lymphangiogenesis and nodal dissemination [249, 250]. TBL1XR1 has been associated with metastatic propensity and appears to participate in lncRNA-regulated dissemination programs, consistent with its role as a transcriptional co-regulator [251]. In HPV-positive cervical carcinoma cells, CDT2/DTL functions within a TRIM22-CDT2-SET8 axis that affects p53 and Rb signaling outputs and supports tumor cell growth and survival [252, 253]. FBXW7 provides a related example of WD40-dependent substrate control, as its mutation or functional disruption can promote proliferation and migration in cervical squamous carcinoma [254]. Translational regulation may also contribute to aggressive behavior, as METTL1 overexpression is associated with poor prognosis and depends on the WD40 cofactor WDR4 to mediate m^7^G tRNA modification [255, 256]. Overall, the current evidence suggests that WDR proteins participate in several CC-relevant programs, including YAP-associated chromatin regulation, RACK1-mediated lymphatic spread, ubiquitin-linked control of p53/Rb-related survival pathways, and WDR4-dependent translational rewiring. Clarifying which of these programs are most informative in defined clinical subgroups may help improve relapse-risk assessment and guide future combination strategies.

#### Renal cell carcinoma

Renal cell carcinoma (RCC), especially clear cell RCC, is characterized by marked spatial and temporal heterogeneity, which continues to complicate diagnosis, prognostic modeling, and treatment selection. Although imaging, immune checkpoint blockade, VEGFR-targeted therapy, and HIF-2α inhibition have improved clinical management, single-site tissue sampling may not fully represent the evolving tumor landscape, and robust biomarkers for choosing or sequencing ICI-based combinations remain limited [275, 276]. Liquid biopsy is also less straightforward in RCC than in several other solid tumors because ctDNA shedding is often low, reducing its sensitivity for early detection and minimal residual disease assessment [277]. In advanced disease, IO/TKI regimens and belzutifan have expanded treatment options, but primary and acquired resistance remain frequent, leaving many post-progression decisions dependent on clinical judgment rather than validated molecular guidance [277, 278]. Against this background, WDR proteins are relevant because several of them intersect with processes that are central to RCC biology, including ribosome production, cell-cycle stress tolerance, chromatin regulation, RNA modification, and immune-associated tumor states (Table 3).

The current RCC literature places WDR proteins within a few biologically coherent processes. BOP1, a WD40 component of the PeBoW ribosome-biogenesis complex, is associated with aggressive ccRCC and poor prognosis, suggesting a role in sustaining biosynthetic and proliferative capacity [257]. WDR43 has been linked to prognosis and immune-related features in kidney cancer cohorts, although its RCC-specific mechanism remains incompletely resolved [203]. Ubiquitin-dependent control is represented by DTL/CDT2 and FBXW7. DTL/CDT2 acts as a CRL4-associated WD40 substrate receptor that supports replication licensing and S-phase stress tolerance, whereas FBXW7 functions as an SCF substrate receptor whose disruption affects oxidative DNA damage control and AKT-related growth signaling [258–260]. Chromatin regulation is reflected by RBBP7, which participates in a BAP1-YY1-associated complex and connects ccRCC driver biology with histone-chaperone function [261]. At the RNA-modification level, WDR4 serves as the WD40 partner of METTL1-mediated m^7^G tRNA modification, linking tRNA function and protein synthesis to oncogenic associations in kidney cancer datasets [262]. In sum, WDR proteins in RCC are best interpreted as components of ribosome-biogenesis, ubiquitin-ligase, chromatin-regulatory, and RNA-modification machinery. Their relevance will become clearer when these mechanisms are tested alongside established clinical variables such as stage, grade, molecular subtype, immune context, and treatment response.

#### Bladder cancer

Bladder cancer (BCa) remains constrained by a high-burden diagnostic pathway and persistent therapeutic uncertainty. In non-muscle invasive disease, frequent long-term surveillance still depends on cystoscopy, while urine based approaches such as urinary tumor DNA can improve detection and monitoring but have not yet been uniformly translated into a standardized, practice changing alternative across clinical contexts [279]. In advanced urothelial carcinoma, many patients cannot receive cisplatin based chemotherapy, and although enfortumab vedotin plus pembrolizumab has set a new first line benchmark, challenges in biomarker guided selection, sequencing, toxicity management, and resistance remain central barriers to durable control [280]. Against this backdrop, WDR protein research offers a rational way to break through these barriers (Table 3).

In bladder cancer (BCa), WDR proteins have been linked to several processes that are directly relevant to invasion, metastasis, and treatment response. WDR54 functions as a proteostasis-related regulator by stabilizing MEMO1 through reduced ubiquitination, thereby enhancing motility signaling, promoting tumorigenesis and metastasis, and decreasing cisplatin sensitivity [147]. WDR5 represents an epigenetic scaffold in BCa. Pharmacological disruption of the WDR5-MLL complex with OICR-9429 suppresses proliferation and metastatic traits, induces apoptosis, attenuates PD-L1-associated immune evasion, and improves cisplatin responsiveness, linking H3K4 methylation machinery to both tumor progression and therapy response [263]. At the post-transcriptional level, WDR4 is elevated in BCa and promotes proliferation and lymph-node metastasis, consistent with a METTL1-WDR4 m7G tRNA modification program that supports translational output in aggressive disease [264]. Ubiquitin-dependent regulation is further reflected by DTL and FBXW7. DTL, a CRL4-associated WD40 substrate receptor, is associated with poor prognosis and promotes malignant progression through AKT/mTOR signaling and cell-cycle-related programs [265]. DDB1 functions as a core CUL4 adaptor in a PRPF19-linked axis that contributes to DNA repair output and gemcitabine response in BCa models [266]. FBXW7 provides a tumor-suppressive counterpart, acting as a WD40-containing F-box substrate receptor that restrains BCa progression by promoting ubiquitination-dependent degradation of ZMYND8 [267]. Overall, these studies suggest that WDR proteins in BCa influence tumor behavior through distinct but connected mechanisms, including MEMO1 stabilization, WDR5-dependent chromatin regulation, WDR4-associated translational rewiring, CRL4-linked cell-cycle and DNA repair control, and FBXW7-mediated degradation of oncogenic substrates. These mechanisms may help explain metastatic risk and chemotherapy response, but their clinical use will require validation in well-defined BCa cohorts and integration with established pathological and molecular features.

### Hematopoietic system tumors

#### Leukemia

Leukemia comprises a diverse group of hematologic malignancies, commonly classified by disease tempo and lineage, including acute or chronic and myeloid or lymphoid forms [281]. Although molecular profiling and measurable residual disease assessment have improved risk evaluation, relapse prediction remains imperfect. Clonal heterogeneity, phenotypic variation, and therapy-driven evolution can make a single diagnostic sample insufficient to capture the full disease landscape. MRD interpretation is also complicated by platform differences, variable thresholds, and persistent mutations that may reflect clonal hematopoiesis rather than residual leukemia [282]. Treatment outcomes have improved, but resistance remains frequent, including in venetoclax-based AML therapy and newer genotype-directed approaches such as menin inhibition [283]. In leukemia, the relevance of WDR proteins is most evident in processes that sustain malignant cell fitness, including transcriptional addiction, ubiquitin-dependent protein turnover, replication-stress adaptation, and targeted protein degradation. (Table 4).

**Table 4:** WDR proteins in hematopoietic system tumors: mechanistic functions and clinical significance

Cancer type	WDR protein	Role	Representative mechanism/pathway	Clinical/translational relevance	Ref
Leukemia	WDR5	Oncogenic	Maintains leukemic transcriptional programs/chromatin states	Target for pharmacologic degradation	[95]
Leukemia	RBBP4	Oncogenic	Maintains leukemic chromatin state; associated with HDAC inhibitor sensitivity when perturbed	Therapy response	[284]
Leukemia	WDR82	Oncogenic	Recruits SETD1A to TSS; NUP98 depends on WDR82-SETD1A axis for H3K4me3 deposition	Aggressive AML programs	[285]
Leukemia	DDB1	Oncogenic/context	Determines molecular-glue behavior; S656 bridges CDK12 and DDB1 to degrade cyclin K	Targeted degradation opportunity	[286]
Leukemia	DCAF15	Oncogenic/dependency	AML-biased dependency; loss impairs replication fork integrity and suppresses AML growth	Replication-stress vulnerability	[287]
Leukemia	DCAF1/VPRBP	Context/therapeutic handle	Resolved DCAF1-PROTAC-WDR5 ternary complexes	Alternative ligase handle for degraders	[94]
Leukemia	FBXW7	Tumor suppressive	Mutations stabilize NOTCH1 intracellular domain in T-ALL	Leukemogenesis/prognosis	[288]
Leukemia	RACK1/GNB2L1	Oncogenic	Promotes AML proliferation partly through GSK3beta-linked signaling	Cell proliferation	[289]
Lymphoma	WDR5	Oncogenic	MYC cofactor on chromatin; inhibitors show single-agent activity in DLBCL models	Actionable dependency	[290]
Lymphoma	WDR4	Oncogenic	METTL1-WDR4 m^7^G tRNA machinery enhances oncogenic mRNA translation	DLBCL tumorigenesis	[291]
Lymphoma	DCAF13	Oncogenic	STAT5B-driven DCAF13 suppresses ferroptosis through p53/xCT pathway	Mantle cell lymphoma progression	[292]
Lymphoma	FBXW7	Context-dependent	Promotes KMT2D degradation in DLBCL; recurrent mutation in CLL reduces NOTCH1 turnover	Survival and sustained NOTCH signaling	[293]

In leukemia, WDR proteins are most clearly connected to chromatin dependency and protein-turnover control. WDR5 is the best-developed example. As a core scaffold of COMPASS/MLL complexes, it helps maintain oncogenic transcription in KMT2A/MLL-rearranged AML, and WIN-site inhibitors or degraders have made WDR5 one of the more advanced WD40-directed targets in this disease context [95]. Other chromatin-associated WDR proteins contribute through more specific mechanisms. RBBP4, a histone-binding WD40 subunit, can be regulated by RNF5-mediated ubiquitination in AML, thereby altering transcriptional programs that support leukemic fitness and influence sensitivity to HDAC inhibitor-based treatment [284]. WDR82 recruits SETD1A-COMPASS to transcription start sites; in NUP98-driven AML, this axis contributes to local H3K4me3 deposition and aberrant gene activation [285]. The ubiquitin system provides another major point of entry. DDB1 acts as a core CRL4 adaptor and is directly relevant to molecular glue pharmacology. The cyclin K degrader S656, for instance, bridges CDK12 to DDB1 and induces cyclin K degradation with anti-AML activity [286]. DCAF15, a WD40-type CRL4 substrate receptor, appears to represent an AML-biased dependency, since its loss impairs replication fork integrity, increases DNA damage, and suppresses AML growth [287]. DCAF1/VPRBP extends this concept into targeted protein degradation, with structural studies showing productive DCAF1-based PROTAC ternary complexes, including DCAF1-PROTAC-WDR5 assemblies [94]. In T-ALL, FBXW7 provides a classic example of disrupted substrate recognition: mutations stabilize the NOTCH1 intracellular domain and enhance NOTCH1 signaling, thereby contributing to T-lineage leukemogenesis [288]. Several WDR proteins are linked more closely to leukemic growth signaling than to chromatin or CRL4 machinery. RACK1/GNB2L1 promotes THP-1 AML cell proliferation, at least in part through GSK3β-associated signaling. WDR54 is elevated in T-ALL, and its depletion reduces leukemic viability and xenograft growth, with reported effects on PDPK1-AKT and ERK pathway readouts [289]. Taken as a whole, the leukemia literature suggests that WDR proteins are most informative when their function is tied to a defined leukemic dependency, such as WDR5-supported transcription in MLL-rearranged AML, DCAF15-associated replication stress control, DDB1-linked molecular glue activity, or FBXW7 loss in NOTCH1-driven T-ALL. This is likely to be more useful than treating WDR expression alone as a general marker of leukemia biology.

#### Lymphoma

Lymphoma remains difficult to diagnose and treat due to its molecular and pathological heterogeneity across subtypes like diffuse large B‑cell lymphoma, follicular lymphoma, mantle cell lymphoma, and T‑cell lymphomas. This variability leads to inconsistent treatment responses, challenges in precise classification, and drug resistance and relapse. Lymphoma cells can lose target antigens, reactivate survival pathways, and evade apoptosis, limiting long-term control. Additionally, current diagnostic methods face technical and interpretive limitations, and emerging precision biomarkers have not yet been fully integrated into clinical practice, hindering accurate risk assessment and treatment customization [294]. In this context, the WDR protein family emerges as a promising axis to address these bottlenecks (Table 4).

In lymphoma, available studies link WD40 repeat proteins to several disease-sustaining programs rather than to a single shared pathway. In diffuse large B-cell lymphoma, WDR5 acts as a chromatin-associated MYC cofactor, and pharmacologic inhibition of its interaction site shows notable single-agent activity in preclinical DLBCL models, suggesting that WDR5-dependent transcriptional maintenance may represent a vulnerability in selected tumors [290]. A separate layer involves translational control. The METTL1-WDR4 m^7^G tRNA modification machinery promotes DLBCL tumorigenesis by enhancing oncogenic mRNA translation in vivo, placing WDR4 as a WD40 cofactor that supports the protein-synthesis demands of lymphoma growth [291]. In mantle cell lymphoma, DCAF13 has been linked to ferroptosis suppression through a STAT5B-dependent program involving the p53 and xCT pathway, indicating a role in redox balance and stress tolerance rather than conventional chromatin regulation [292]. Ubiquitin-dependent substrate control is represented by FBXW7. In DLBCL, FBXW7 can promote lymphoma cell survival by facilitating degradation of the chromatin modifier KMT2D, whereas recurrent FBXW7 mutations in chronic lymphocytic leukemia reduce NOTCH1 turnover and sustain NOTCH signaling [293]. These findings suggest that lymphoma-associated WDR proteins should be interpreted according to their functional context, including MYC-linked transcription, m7G-dependent translation, ferroptosis control, and substrate-specific ubiquitin signaling. Their therapeutic relevance is likely to be greatest in molecularly defined settings where these WDR-linked processes are demonstrably required for malignant cell survival or treatment resistance.

### Nervous system tumors

#### Gliomas

Diffuse gliomas, including glioblastoma, remain difficult to diagnose and treat because tumor biology outstrips routine clinical resolution. Diagnosis increasingly depends on integrated molecular profiling, yet limited access to molecular testing can leave a substantial fraction of CNS tumors without a definitive WHO CNS5 assignment, undermining risk stratification and treatment planning [295]. Liquid biopsy approaches are promising for real-time disease monitoring, but in glioma they still face sensitivity and standardization constraints, with CSF ctDNA generally outperforming plasma in detection [296]. Therapeutically, recurrence and resistance dominate outcomes, and immunotherapy benefit is often blunted by profound intratumoral heterogeneity and an immunosuppressive microenvironment [297]. Studies of WDR proteins in glioma offer a promising avenue to help overcome these diagnostic and therapeutic bottlenecks in the future (Table 5).

**Table 5:** WDR proteins in nervous system tumors: mechanistic functions and clinical significance

Cancer type	WDR protein	Role	Representative mechanism/pathway	Clinical/translational relevance	Ref
Gliomas	WDR5	Oncogenic	Sustains GSC transcription via WRAD-SET1/MLL H3K4 methylation	Stemness/CSC programs	[8]
Gliomas	RBBP4	Oncogenic	Upregulates MRN complex and enhances DSB repair after TMZ/irradiation	Chemoradiotherapy resistance	[298]
Gliomas	DDB1/CRL4 axis	Oncogenic/context	CRL4 pathway activation implicated in TMZ resistance	Proteostasis-linked resistant state	[299]
Gliomas	FBXW7	Context-dependent	Deficiency creates DNA repair and redox liabilities in IDH1-mutant glioma	Radiotherapy response biomarker	[300]
Gliomas	WDR1	Oncogenic	Facilitates cofilin-dependent actin remodeling	Aggressive/invasive phenotype	[301]

In glioma, WDR proteins are most relevant when viewed against the biological features that drive recurrence, including stem-like transcriptional programs, DNA repair capacity, therapy-induced proteostasis, and diffuse invasion. WDR5 acts as a chromatin scaffold in glioblastoma cancer stem cells, where it supports WRAD-SET1/MLL-dependent H3K4 methylation and sustains stemness-associated transcriptional output; WDR5 inhibition disrupts WRAD assembly, reduces H3K4me3, and suppresses CSC programs [8]. RBBP4, a histone-binding WD40 factor, links chromatin regulation to treatment resistance by transcriptionally upregulating the MRN complex and enhancing double-strand break repair after TMZ and irradiation [298]. Ubiquitin-mediated proteostasis also appears relevant to glioma therapy failure. CRL4 pathway activation, including CUL4B-associated mechanisms, has been implicated in TMZ resistance, suggesting that DDB1-centered CRL4 machinery may help maintain drug-tolerant states, although this axis still requires more direct glioma-specific validation [299]. FBXW7 offers a genetically anchored example of WD40-dependent substrate control. As an F-box substrate receptor, loss of FBXW7 exposes IDH1-mutant glioma cells to DNA-repair and redox stress, thereby increasing radiosensitivity [300]. WDR1 further links WD40 biology to the invasive phenotype of glioblastoma. By facilitating cofilin-dependent actin remodeling, WDR1 has been associated with aggressive primary glioblastoma, and recent chemical biology studies suggest that WDR1-associated complexes may be exploitable regulators of cytoskeletal dynamics and invasion [301].Overall, these findings indicate that WDR proteins can capture clinically important glioma states such as stemness, repair competence, proteostasis dependence, and invasive potential, thereby offering state-aligned biomarkers to refine diagnosis and risk stratification. At the same time, they highlight combinable vulnerabilities that could be exploited to sensitize tumors to TMZ or radiotherapy and to limit invasion-driven recurrence, supporting more rational, biomarker-guided treatment strategies.

### Endocrine system tumors

#### Thyroid cancer

Thyroid cancer care is still limited by diagnostic ambiguity in a substantial subset of patients and by therapy resistance in advanced disease. A persistent diagnostic bottleneck is the large proportion of cytologically indeterminate thyroid nodules, where even widely used molecular classifiers show variable accuracy across platforms and real-world settings, leaving residual uncertainty in malignancy risk and surgical decision-making [302]. Therapeutically, outcomes worsen sharply once tumors become radioiodine-refractory, and although MAPK-pathway “redifferentiation” can restore radioiodine uptake and clinical responses in a subset, consistent patient selection and durable control remain unmet needs [303]. At the aggressive extreme, anaplastic thyroid carcinoma continues to progress rapidly with frequent resistance even as targeted and immunotherapy combinations are being explored [303]. These bottlenecks highlight the need for new state-aligned biomarkers and actionable targets, and WDR proteins are well positioned to help improve thyroid cancer diagnosis and guide more effective, combinable treatment strategies to reduce relapse (Table 6).

**Table 6:** WDR proteins in endocrine system tumors: mechanistic functions and clinical significance

Cancer type	WDR protein	Role	Representative mechanism/pathway	Clinical/translational relevance	Ref
Thyroid cancer	WDR5	Oncogenic	Promotes proliferation and migration; associated with aggressive features	Poor survival/prognosis	[304]
Thyroid cancer	FBXW7	Tumor suppressive	FBXW7 restrains papillary thyroid cancer progression and counteracts FOXP4-driven malignant phenotypes	Higher expression associated with favorable prognosis	[305]
Thyroid cancer	FBXW10	Oncogenic	Pro-growth effector driven by upstream transcriptional activation	Proliferation/tumor growth	[306]
Thyroid cancer	WDR4	Oncogenic	METTL1-WDR4-linked m^7^G translational control drives progression and metastasis	Metastatic potential	[307]
Thyroid cancer	WDR77	Predisposition factor	Loss-of-function variants predispose to familial nonmedullary thyroid cancer	Germline risk	[308]
Thyroid cancer	RACK1	Oncogenic/context	Ras-Raf pathway-related signaling hub	Signaling hub candidate	[309]

In thyroid cancer, a diverse set of WDR proteins has been implicated across tumor initiation risk, epigenetic state control, proteostasis, and translational output. In papillary thyroid carcinoma, WDR5 is associated with aggressive clinicopathologic features and poorer survival, and functional studies support its role in promoting proliferation and migration, consistent with a chromatin-scaffold function in oncogenic transcriptional regulation [304]. FBXW7 acts through a tumor-suppressive substrate-recognition mechanism. In the FOXP4-FBXW7 regulatory axis, higher FBXW7 expression is associated with more favorable outcomes, whereas restoration of FBXW7 counteracts malignant phenotypes, supporting its role as a WD40-containing F-box substrate receptor rather than a downstream signaling effector [305]. FBXW10, another F-box WD40 protein, appears to have an opposite tumor-promoting role, as transcriptional activation of FBXW10 enhances thyroid cancer proliferation and tumor growth in model systems, although its substrate specificity remains less clearly defined [306]. At the post-transcriptional level, WDR4 functions as the WD40-containing cofactor of METTL1-mediated m^7^G tRNA modification, and this module has been linked to papillary thyroid cancer progression and metastasis through codon-biased translational control [307]. Germline genetics further supports a role for WD40 biology in thyroid tumor predisposition, with loss-of-function variants in WDR77 reported to predispose to familial nonmedullary thyroid cancer [308]. RACK1/GNB2L1 further extends WD40 biology to signaling organization through its proposed involvement in Ras-Raf pathway regulation in papillary thyroid cancer [309]. Overall, these WD40 proteins in thyroid cancer matter because they can sharpen diagnosis-oriented stratification and inform treatment planning. They offer biology-based markers to refine risk, prognosis, and metastatic potential beyond stage alone, and they highlight pathway-linked dependencies in chromatin control, translation, and ubiquitin-mediated proteostasis that may be leveraged for biomarker-guided therapy selection and combination strategies to improve response durability.

### Integumentary system tumors

#### Cutaneous melanoma

Melanoma care has improved with dermoscopic assessment, immune checkpoint blockade, and BRAF/MEK targeted therapy, but earlier diagnosis and durable disease control remain uneven. Screening is limited by imperfect specificity, resulting in biopsy burden, and aggressive subtypes such as acral and amelanotic melanoma are often detected late [310]. Algorithm-assisted assessment may improve diagnostic performance, but its real-world use still depends on generalizability, workflow integration, and prospective validation [311]. In advanced disease, response heterogeneity, treatment-related toxicity, and primary or acquired resistance continue to limit the long-term benefit of immunotherapy and MAPK-targeted therapy [312]. In this setting, WDR proteins may be relevant because they influence signaling, transcriptional regulation, and proteostasis under therapeutic pressure, potentially helping to define resistance-prone tumor states and combination-ready vulnerabilities (Table 7).

**Table 7:** WDR proteins in endocrine system tumors: mechanistic functions and clinical significance

Cancer type	WDR protein	Role	Representative mechanism/pathway	Clinical/translational relevance	Ref
Cutaneous melanoma	FBXW7	Tumor suppressive	Loss drives sustained NOTCH1 activation	Pathway-matched vulnerability	[313]
Cutaneous melanoma	DTL/CDT2	Oncogenic	CRL4-CDT2-SET8/p21 axis disrupted by pevonedistat	Therapeutically actionable, synergy with BRAF inhibition	[314]
Cutaneous melanoma	RACK1	Oncogenic	Upregulated scaffold linked to tumorigenicity and clinical outcome	Growth/outcome	[315]
Cutaneous melanoma	WDR26	Oncogenic	lncRNA-regulated shift in subcellular interactions promotes AKT signaling and invasion	Metastatic signaling node	[316]
Cutaneous melanoma	DCAF1/VprBP	Oncogenic	Phosphorylates histone H2A Thr120 to silence growth-regulatory genes	Chromatin-control node	[317]
Cutaneous melanoma	WDR5	Context-dependent	Participates in BRD4/lncRNA NEAT1-related transcriptional tuning	Chromatin-dependent gene expression state	[318]

Compared with some other cancers, the melanoma literature on WDR proteins remains relatively focused, but several examples are mechanistically informative. FBXW7, a WD40-containing F-box substrate receptor, is recurrently mutated or inactivated in melanoma, and its loss sustains NOTCH1 activation, suggesting that NOTCH signaling may be particularly relevant in FBXW7-deficient tumors [313]. DTL/CDT2 represents another ubiquitin-linked factor. As the substrate receptor of the CRL4-CDT2 complex, DTL regulates SET8 and p21 ubiquitylation and degradation. Inhibition of neddylation with pevonedistat disrupts this CRL4-CDT2-dependent circuit, suppresses melanoma growth in vitro and in vivo, and shows synergy with BRAF inhibition in BRAF-mutant models [314]. RACK1/GNB2L1 has been reported to be upregulated in melanoma lesions and associated with tumorigenicity and clinical outcome, consistent with its role as a signaling scaffold that supports growth-promoting pathways [315]. WDR26 has also been linked to invasive behavior through lncRNA-regulated changes in subcellular interactions that enhance AKT signaling, placing it close to metastasis-related signal organization [316]. DCAF1, also known as VprBP, is highly expressed in melanoma cells and acts as an epigenetic effector by phosphorylating histone H2A at Thr120, which promotes transcriptional silencing of growth-regulatory genes and supports melanomagenic programs, positioning DCAF1 as a WD40-linked chromatin-control node with therapeutic implications [317]. WDR5 is implicated in RNA-guided transcriptional regulation, as NEAT1 engages BRD4 and modulates BRD4-associated transcriptional output involving BRD4/WDR5 target genes [318].Together, these findings suggest that WDR proteins in melanoma can improve future care by providing state-aligned biomarkers for risk and response stratification and by revealing actionable pathway dependencies. In particular, WDR-linked nodes in NOTCH signaling, CRL4-driven proteostasis, and chromatin regulation support more rational, biomarker-guided combinations aimed at reducing resistance and relapse.

### Musculoskeletal system tumors

#### Osteosarcoma

Osteosarcoma care is still limited by imprecise risk stratification at diagnosis and poor durable control after relapse. Outcomes have largely plateaued for decades, and metastatic or recurrent disease remains hard to cure due to early dissemination, intratumoral heterogeneity, and chemoresistance despite multi-agent chemotherapy and surgery [319]. In parallel, attempts to extend benefit with targeted therapy and immunotherapy have been hindered by pathway redundancy, microenvironmental constraints, and inconsistent clinical efficacy, leaving relapse management largely empirical [320]. From a diagnostic and monitoring perspective, there is strong interest in noninvasive biomarkers for early relapse and treatment response, yet liquid biopsy approaches in sarcoma, including osteosarcoma, still face sensitivity and standardization hurdles that limit routine clinical integration [321]. These bottlenecks highlight WDR proteins as a promising avenue to improve osteosarcoma diagnosis and therapy by enabling more informative biomarkers and revealing combinable therapeutic vulnerabilities that may help reduce relapse (Table 8).

**Table 8:** WDR proteins in musculoskeletal system tumors: mechanistic functions and clinical significance

Cancer type	WDR protein	Role	Representative mechanism/pathway	Clinical/translational relevance	Ref
Osteosarcoma	WDR5	Oncogenic	Cell geometry/contractility promote nuclear WDR5 activity, increasing H3K4me3 and self-renewal factors	Stemness-supporting axis	[322]
Osteosarcoma	WDR4	Oncogenic	METTL1-WDR4 overexpression drives codon-biased translation and doxorubicin resistance	Drug resistance/translation control	[323]
Osteosarcoma	WDR1	Oncogenic	Downregulation reduces migration/invasion, consistent with actin-remodeling dependency	Metastatic competence	[324]
Osteosarcoma	FBXW7	Tumor suppressive	Reduced expression associated with adverse outcomes and therapy resistance	Proteostasis-based stratification	[325]
Osteosarcoma	WDR74	Oncogenic/context	Implication in growth and migration downstream of ncRNA/RNA-modification pathways	Growth and migration	[326]
Osteosarcoma	WDR3	Oncogenic/biomarker-like	Forms liquid-like condensates; nilotinib suppresses phenotypes partly by inhibiting condensate formation	Prognostic/treatment response biomarker candidate	[327]

In osteosarcoma, WDR-related studies have begun to connect these proteins with several disease-relevant processes. WDR5 functions as a chromatin-associated regulator of stem-like behavior. Mechanical cues from cell geometry and contractility can enhance nuclear WDR5 activity, increase H3K4me3, and upregulate self-renewal factors, suggesting that WDR5-dependent chromatin output helps translate microenvironmental signals into stemness programs [322]. At the post-transcriptional level, the METTL1-WDR4 complex is overexpressed in osteosarcoma and promotes codon-biased translation, tumorigenic behavior, and doxorubicin resistance, placing WDR4 as a WD40-containing cofactor in m^7^G-linked translational control [323]. WDR1 is more closely related to invasion, as its downregulation reduces osteosarcoma cell migration and invasion, consistent with a role in cofilin-dependent actin remodeling and metastatic potential [324]. FBXW7 represents the tumor-suppressive arm of WD40 substrate recognition. Reduced FBXW7 expression is associated with adverse outcomes, and its suppression contributes to therapy resistance in preclinical models, linking defective ubiquitin-mediated proteostasis to aggressive disease behavior [325]. Emerging RNA-regulatory work has begun to implicate additional WDR family members such as WDR74 in osteosarcoma growth and migration programs downstream of noncoding RNA and RNA-modification pathways, expanding the WDR landscape beyond classic chromatin and ubiquitin nodes [326]. WDR3 adds a newer layer of regulation through phase separation. WDR3 forms liquid-like condensates in osteosarcoma cells, and nilotinib suppresses osteosarcoma phenotypes in part by disrupting WDR3 condensate formation, suggesting that condensate biology may contribute to tumor growth and treatment response [327]. Collectively, these osteosarcoma studies suggest that WDR proteins could support future care by providing state-aligned biomarkers for aggressive, metastatic, and drug-resistant disease and by revealing actionable dependencies in chromatin control, m^7^G-linked translation, cytoskeletal invasion, proteostasis, and condensate biology. This creates a rationale for more precise risk stratification and for mechanism-guided combinations aimed at improving chemotherapy response and reducing relapse.

## Cross-system commonalities and differences

### Commonalities

Across diverse cancers, the literature summarized above supports a recurrent involvement of WDR proteins in malignant progression through several conserved regulatory layers, with context-dependent emphasis that differs by tissue lineage, microenvironmental constraints, and treatment pressure. Chromatin-associated regulation is a frequent entry point. WD40 proteins such as WDR5 and other WD40-containing chromatin components support oncogenic transcriptional output and can be directly interrogated pharmacologically. Structure-guided development of potent WDR5 WIN-site inhibitors with in vivo efficacy illustrates that selected WD40 interaction pockets are tractable drug targets and provides a framework for testing whether disrupting WDR5-dependent transcriptional programs can enhance treatment response in multiple settings [165]. Polycomb biology adds a related WD40 dimension through EED, a PRC2 subunit with WD40 repeats. Allosteric EED inhibition has been shown to suppress tumor progression in part by modulating immune-relevant features of the tumor microenvironment, supporting the view that epigenetic WD40 nodes can influence both cancer cell intrinsic transcription and host antitumor responses [168, 328].

Translation and RNA modification represent another cross-cancer layer that appears repeatedly across organ systems. The METTL1–WDR4 complex installs tRNA m^7^G modifications and can bias translational output toward growth and invasion programs, with mechanistic evidence in lung cancer linking METTL1/WDR4 to proliferation, invasion, and in vivo tumorigenicity [45]. This type of translational rewiring aligns with clinical phenotypes that recur across solid tumors, including EMT-like plasticity and chemotherapy resistance, and it provides a mechanistic basis for incorporating WDR4-linked readouts into response and relapse risk models in multiple tumor types. Ubiquitin-dependent proteostasis is also a recurring layer, particularly through WD40 substrate receptors and adaptors in SCF and CRL4 complexes. Reviews of the CRL4^Cdt2^ axis emphasize its central role in coupling DNA replication and genome stability programs to regulated proteolysis, providing a unifying explanation for why DTL/CDT2- and DDB1-centered circuits repeatedly emerge in studies of therapy response, replication stress tolerance, and resistance evolution [329]. A parallel, widely observed theme is the tumor-suppressive role of the WD40 F-box receptor FBXW7, which constrains diverse oncogenic substrates and is frequently altered across cancers, thereby linking impaired degron surveillance to treatment failure and aggressive biology [6].

Additional commonalities expand the cross-system picture beyond chromatin, translation, and proteostasis. Cytoskeletal regulation and mechanotransduction appear in several epithelial cancers, with WDR1 connected to actin remodeling and YAP signaling in NSCLC, a mechanism that maps directly onto invasion and metastasis-relevant phenotypes [330]. Arginine methylation-linked transcriptional control provides another conserved axis via PRMT5 and its WD40 cofactor WDR77 (MEP50), where the TGFβ–PRMT5–MEP50 pathway has mechanistic evidence for regulating invasion programs, supporting the inclusion of WD40-associated methylation modules when comparing metastatic competence across tumor types [49]. Ribosome biogenesis and nucleolar growth programs repeatedly feature in digestive and urogenital malignancies and also appear in other contexts through PeBoW-related biology that includes the WD40 protein WDR12; this is consistent with a broad reliance on elevated biosynthetic capacity and nucleolar stress tolerance during malignant progression [331]. Telomere and genome maintenance mechanisms add a further WD40-relevant layer through WRAP53β (TCAB1/WDR79), which supports Cajal body integrity, telomerase trafficking, and DNA damage response functions, providing a mechanistic bridge between WD40 scaffolding and immortality and repair competence across cancers [332]. Finally, emerging work in mesenchymal tumors suggests that noncanonical regulatory modes may matter in selected settings, exemplified by WDR3 phase separation biology linked to therapeutic response in osteosarcoma [327].

### Differences

Differences between systems are best interpreted as shifts in which WDR-linked layer most strongly aligns with the dominant clinical bottleneck of a given tumor type. In lung cancer, cytoskeletal and mechanotransduction wiring can be prominent and directly related to invasive behavior and metastasis risk [170]. In digestive system tumors, nucleolar and ribosome biogenesis outputs commonly stand out alongside proteostasis and EMT-related chromatin remodeling, consistent with high biosynthetic demand and stress adaptation as key drivers of relapse and treatment variability [192]. In nervous system tumors, repair competence and infiltrative recurrence elevate the relevance of chromatin-facing and genome maintenance-associated WDR mechanisms, which can shape chemoradiotherapy resistance [298]. In hematologic malignancies, WDR related dependencies often map to epigenetic control and replication stress associated programs, and WDR5 provides a representative example. Potent WIN site inhibitors can displace WDR5 from chromatin and suppress WDR5 linked transcriptional output, and genetic evidence supports an essential role of WDR5 in MLL rearranged leukemogenesis, together supporting the feasibility of targeting WDR5 in leukemia relevant contexts [333].

Overall, these comparisons support organizing this section around shared mechanistic layers, including chromatin regulation, translation and RNA modification, ubiquitin mediated proteostasis, cytoskeletal control, and ribosome biogenesis, while emphasizing that the most informative WDR candidates and the most testable intervention hypotheses remain tumor type dependent and should be prioritized according to context specific failure modes and treatment pressures.

## Clinical trials in cancer therapy

Current translational progress for WDR proteins is most clinically advanced where a clearly druggable WD40 pocket drives a disease-critical epigenetic switch, exemplified by the PRC2 subunit EED, a canonical WD40 β-propeller that recognizes H3K27me3 and allosterically activates PRC2. Multiple orally bioavailable, allosteric EED inhibitors that occupy the EED–H3K27me3 binding cavity have entered early-phase clinical development: MAK683 has completed first-in-human phase 1/2 evaluation in advanced malignancies (NCT02900651), establishing human PK/PD feasibility and preliminary antitumor activity consistent with PRC2 pathway suppression [334]. In parallel, additional EED-targeting clinical candidates include APG-5918 for advanced solid tumors/lymphomas (NCT05415098) [60] and ORIC-944 (rinzimetostat) for metastatic prostate cancer (NCT05413421) [335], reinforcing EED as a clinically testable “WD40 pocket” paradigm in oncology. Beyond cancer, EED inhibition is also being explored in hemoglobinopathies via FTX-6058 (pociredir), a selective EED inhibitor evaluated for fetal hemoglobin induction in sickle cell disease (NCT05169580), illustrating that WD40-targeted epigenetic modulation can extend to non-oncology indications [336]. Alongside these clinical efforts, a broad preclinical wave is expanding the druggability of WDR proteins across oncology-relevant scaffolds. WDR5, a WD40 β-propeller that sits at the core of chromatin and transcriptional assemblies, has an increasingly mature inhibitor landscape, particularly through WIN-site antagonists. Across multiple cancer models, including neuroblastoma, these compounds have been supported by substantial mechanistic validation and pharmacology optimization reported in recent syntheses and primary studies, collectively strengthening the rationale for advancing WDR5 programs toward clinical-grade antagonists [75, 337]. Finally, non-epigenetic WDR scaffolds are also being pharmacologically probed at the preclinical stage; for example, RACK1 (a WD40-repeat scaffold) has been inhibited using a function-blocking antibody approach that suppresses tumor cell proliferation, underscoring the feasibility of targeting WD40 scaffolds through modalities beyond small molecules when appropriate epitopes and mechanisms are defined [338].

Although WDR proteins have been extensively implicated in tumor initiation, progression, metastasis, immune regulation, and therapy resistance, their clinical translation remains highly uneven [3]. A major reason is that most WDR proteins are non-enzymatic scaffolds rather than classical catalytic targets. Therefore, successful therapeutic development does not simply depend on suppressing enzymatic activity, but on identifying structurally defined interaction pockets, disease-critical protein–protein interfaces, or tumor-state dependencies that can be pharmacologically disrupted [339]. From this perspective, the current clinical landscape of WDR-targeted cancer therapy can be divided into two major categories: clinically advanced WD40-pocket inhibitors, represented by EED-directed PRC2 inhibitors (NCT02900651) [334], and highly promising but still largely preclinical WDR-directed strategies, represented by WDR5 inhibitors and degraders [340].

Among WDR family members, EED provides the clearest example of why certain WDR proteins have advanced into clinical development. EED is a WD40 β-propeller subunit of PRC2 that recognizes H3K27me3 through a defined aromatic cage and allosterically activates EZH1/2-mediated H3K27 methylation. This creates three advantages for drug development: a structurally tractable binding pocket, a direct pharmacodynamic marker through H3K27me3 reduction, and a biologically coherent dependency in tumors driven by PRC2 activity [60, 65]. These features collectively explain why EED inhibitors have progressed more rapidly than many other WDR-targeted agents. MAK683, an orally available allosteric EED inhibitor, has been evaluated in a first-in-human phase 1/2 dose-escalation study in patients with advanced malignancies. In this study, 139 patients were treated, H3K27me3 reduction in peripheral blood mononuclear cells and tumor biopsies supported PRC2 pathway modulation, and preliminary clinical activity was observed in diffuse large B-cell lymphoma and epithelioid sarcoma [334]. In parallel, APG-5918 is being evaluated in a multicenter, open-label phase 1 dose-escalation and expansion study in patients with advanced solid tumors or lymphomas (NCT05415098) [341]. ORIC-944, also known as rinzimetostat, is being developed as a potent, selective, orally bioavailable allosteric PRC2 inhibitor through EED binding, and is currently being assessed as monotherapy or in combination with androgen receptor pathway inhibitors in metastatic prostate cancer (NCT05413421) [342]. Together, these clinical programs establish EED inhibition as the most mature paradigm for directly drugging a WD40-containing epigenetic reader in oncology (Table 9).

**Table 9:** Translational maturity, biomarker logic and clinical-development challenges of WDR-targeted therapeutic strategies in cancer

WDR target or WDR-associated therapeutic axis	Representative agents or strategies	Mechanistic basis for therapeutic targeting	Clinical development stage	Disease context and trial rationale	Biomarker and pharmacodynamic considerations	Major translational challenges
EED, PRC2 WD40 reader subunit	MAK683	Allosteric inhibition of the EED aromatic cage disrupts H3K27me3-dependent PRC2 activation and suppresses Polycomb-mediated transcriptional repression	First-in-human phase 1/2 study reported	Advanced solid tumors and hematologic malignancies, with preliminary activity observed in PRC2-dependent contexts such as diffuse large B-cell lymphoma and epithelioid sarcoma	H3K27me3 reduction in tumor tissue or peripheral blood cells; PRC2 pathway activity; SWI/SNF deficiency; Polycomb-dependent transcriptional states	Limited efficacy in molecularly unselected populations; incomplete PRC2 suppression; compensatory chromatin rewiring; need for response-enriched patient selection
EED, PRC2 WD40 reader subunit	APG-5918	Oral allosteric EED inhibition designed to suppress PRC2-dependent H3K27 methylation and downstream oncogenic transcriptional programs	Phase 1 dose-escalation and expansion study	Advanced solid tumors and lymphomas, particularly tumors with epigenetic dependence or refractory disease biology	H3K27me3 pharmacodynamic suppression; PRC2 dependency signatures; tumor lineage and epigenetic vulnerability markers	Predictive biomarkers remain immature; optimal combination partners are unclear; adaptive escape through alternative epigenetic programs may occur
EED, PRC2 WD40 reader subunit	ORIC-944, rinzimetostat	Selective oral allosteric PRC2 inhibition through EED binding, intended to counteract PRC2-driven lineage plasticity and therapy escape	Phase 1/1b clinical development	Metastatic prostate cancer, as monotherapy or combined with androgen receptor pathway inhibitors	PRC2 activity; H3K27me3 dynamics; androgen receptor inhibitor resistance; lineage plasticity and neuroendocrine transition markers	Need to define rational sequencing and combination strategies; possible lineage switching, neuroendocrine differentiation, and activation of bypass survival pathways
WDR5, chromatin scaffold within SET1/MLL complexes	WIN-site inhibitors, including OICR-9429 and next-generation analogues	Disruption of the WDR5 WIN-site interface interferes with selected WDR5-dependent chromatin interactions and oncogenic transcriptional programs	Preclinical	MLL-rearranged leukemia, MYC-driven tumors, and selected solid tumors with WDR5-dependent transcriptional addiction	WDR5 chromatin occupancy; MYC-associated transcriptional output; MLL-fusion dependency; sensitivity signatures identified by functional profiling	WIN-site blockade affects only part of WDR5 biology; non-WIN interactions may persist; therapeutic window and patient-selection criteria remain unresolved
WDR5, chromatin scaffold protein	MS33, MS67 and other WDR5-directed degraders	Targeted degradation removes WDR5 protein rather than inhibiting a single interaction interface, potentially suppressing broader WDR5 scaffold functions	Preclinical	MLL-rearranged acute myeloid leukemia, pancreatic cancer, and other WDR5-dependent tumor models	WDR5 protein depletion; suppression of WDR5-regulated transcriptional networks; c-MYC and MLL complex outputs; degrader-sensitive tumor states	Pharmacokinetic optimization, tissue penetration, E3 ligase compatibility, degradation durability, and toxicity from global WDR5 loss require further validation
PRMT5-WDR77, MEP50 methyltransferase complex	PRMT5 inhibitors; MTA-cooperative PRMT5 inhibitors in MTAP-deleted tumors	Pharmacological inhibition targets PRMT5 enzymatic activity within a WDR77-containing methyltransferase complex, indirectly suppressing WDR-associated arginine methylation programs	Clinical development of PRMT5 inhibitors ongoing; WDR77 itself is not directly targeted	Hematologic malignancies and selected solid tumors, especially tumors with MTAP deletion or PRMT5-dependent splicing vulnerability	MTAP deletion; MTA accumulation; PRMT5 dependency; splicing perturbation; symmetric arginine dimethylation levels	This represents indirect targeting of a WDR-associated complex rather than direct WDR inhibition; the specific contribution of WDR77 remains difficult to isolate clinically
DCAF and FBXW family substrate receptors	Molecular-glue-like strategies, degrader platforms, downstream pathway-matched combinations	WD40 substrate receptors confer substrate specificity to CRL or SCF ubiquitin ligases and may be exploited to remodel oncogenic proteostasis	Mostly exploratory or preclinical	Tumors driven by ubiquitin-dependent degradation defects, altered substrate stability, or therapy-induced proteostatic adaptation	Substrate accumulation or depletion; CRL or SCF activity; degron status; pathway-specific dependency markers	Direct druggability is limited by broad scaffold functions; substrate specificity and context dependence complicate therapeutic design
RACK1, WDR48, WDR43, WDR76 and other scaffold WDR proteins	Interface inhibitors, targeted degraders, RNA-based strategies, or pathway-level combinations	These WDR proteins regulate metastasis, DNA damage response, autophagy, ribosome biogenesis, deubiquitination, and therapy resistance through scaffold-dependent protein complex assembly	Preclinical or discovery stage	Tumor-type-specific contexts involving invasion, stress tolerance, immune evasion, metabolic adaptation, or drug resistance	Tumor-state dependency; target engagement assays; downstream pathway readouts; omics-defined vulnerability signatures	Lack of clearly defined druggable pockets; multifunctional scaffold biology; uncertain therapeutic window; insufficiently validated patient-selection biomarkers

The clinical progress of EED inhibitors also illustrates the importance of rational trial design. Early-phase studies should not merely ask whether a WDR-containing target can be inhibited, but whether target engagement can be linked to a defined epigenetic dependency and a responsive tumor state. For EED inhibitors, this requires integration of target-engagement assays, especially H3K27me3 suppression, because EED recognizes H3K27me3 through an aromatic cage and EED-pocket inhibitors can directly disrupt PRC2 allosteric activation [93, 343]. This pharmacodynamic rationale has been supported clinically by the first-in-human MAK683 study, in which H3K27me3 reduction in peripheral blood mononuclear cells and tumor biopsies indicated PRC2 pathway modulation [334]. Tumor features that may enrich for PRC2 dependence include SWI/SNF complex deficiency, particularly SMARCB1-, ARID1A-, or SMARCA4-deficient contexts, EZH2 pathway activation, and PRC2-dependent transcriptional states [344, 345]. In prostate cancer, the development of ORIC-944 in combination with androgen receptor pathway inhibitors reflects a clinically relevant combination strategy, because EZH2/EED-containing PRC2 has been linked to androgen receptor signaling, castration resistance, and lineage-state remodeling after androgen receptor pathway blockade [342, 346, 347]. Thus, EED inhibitor trials are beginning to move from broad all-comer dose-escalation studies toward pharmacodynamic biomarker-guided, tumor-state-informed, and combination-based development.

By contrast, the clinical development of WDR5-directed therapy remains relatively immature, although its biological rationale is compelling. WDR5 acts as a key chromatin scaffold in SET1/MLL complexes and is also closely linked to MYC-associated transcriptional programs [76, 290]. Preclinical studies have shown that WIN-site inhibitors can interfere with selected WDR5-dependent interactions, reduce WDR5 occupancy on chromatin, and attenuate oncogenic transcriptional programs [92]. More recently, WDR5-targeting degraders, exemplified by MS67, have provided an alternative strategy by removing the scaffold protein itself rather than inhibiting only one interaction interface, thereby potentially overcoming some limitations of conventional small-molecule blockade [95, 348]. Nevertheless, WDR5-targeted approaches have progressed more slowly than EED inhibitors. One important reason is that WDR5 exerts its functions through multiple binding surfaces, so WIN-site inhibition may not be sufficient to abrogate all tumor-promoting activities [339, 349]. In addition, because WDR5 participates in fundamental transcriptional and chromatin-regulatory processes, the therapeutic window and possible lineage-specific toxicities remain key concerns [75]. Another unresolved issue is patient selection. Compared with PRC2-dependent tumors, clinically actionable biomarkers for WDR5 dependency are still poorly defined. Tumor settings such as MLL-rearranged leukemia, MYC-driven cancers, or malignancies with pronounced WDR5-dependent transcriptional addiction may be more responsive, but these indications still require clearer biomarker frameworks and prospective validation before WDR5-directed therapies can be effectively translated into clinical practice [350, 351].

The contrast between EED and WDR5 also points to a broader issue in translating WDR biology into cancer therapy. For a WDR protein to move beyond preclinical interest, it is usually not enough to show that it is overexpressed or functionally involved in tumor progression [352, 353]. Successful clinical development generally requires a druggable structural feature, such as a defined pocket or a dominant interaction surface that can be targeted by a small molecule or degrader [3, 354]. It also requires evidence that tumor cells are truly dependent on that WDR protein in a particular molecular or lineage state, rather than simply showing an association between WDR expression and malignant behavior [352]. Equally important, clinical trials need biomarkers that can verify target engagement and enrich for patients most likely to respond [355, 356].When these elements are absent, WDR-directed agents may remain mechanistically appealing but difficult to advance clinically [357]. This helps explain why many WDR proteins that have been implicated in cancer biology, including DCAF family substrate receptors, RACK1 [236], WDR77/MEP50 [358], WDR48 [359], WDR43 [203], and WDR76 [360], have not yet produced mature therapeutic programs. Their oncogenic functions may be well supported in experimental models, but in many cases the actionable interface, the most suitable therapeutic strategy, and the biomarker framework for patient selection are still not sufficiently defined.

Emerging resistance mechanisms should also be considered early in WDR-targeted clinical development. For EED inhibitors, resistance may arise through incomplete PRC2 suppression, compensatory EZH1/2-dependent or independent chromatin pathways, changes in PRC2 subunit composition, persistence or restoration of H3K27me3 at critical loci, or lineage-state switching that bypasses Polycomb dependence [65, 361]. In combination settings, such as PRC2 inhibition plus androgen receptor pathway blockade, tumors may escape through alternative lineage programs, neuroendocrine differentiation, or activation of parallel survival pathways [362, 363]. For WDR5-directed agents, potential resistance mechanisms may include mutations or conformational changes at drug-binding interfaces, compensatory recruitment of alternative chromatin scaffolds, incomplete disruption of non-WIN interactions, or adaptive rewiring of MYC- and MLL-associated transcriptional networks [351, 364]. These possibilities argue for longitudinal pharmacodynamic profiling, paired tumor biopsies when feasible, circulating tumor DNA monitoring, and transcriptomic or epigenomic state tracking during treatment [355, 365].

Taken together, the clinical development of WDR-targeted therapy is entering an early transition phase. EED inhibitors have shown that WD40 β-propeller proteins can be pharmacologically engaged in humans when a defined structural pocket is linked to a biologically plausible and clinically testable tumor dependency. WDR5 inhibitors and degraders further illustrate how next-generation strategies may extend WDR targeting beyond classical pocket-based inhibition toward interface disruption and targeted protein degradation. Future progress will depend on moving from target nomination to state-matched clinical development, in which drug modality, tumor context, pharmacodynamic biomarkers, and rational combinations are integrated from the outset. Such a framework will be essential for transforming WDR proteins from mechanistic cancer regulators into clinically actionable therapeutic vulnerabilities.

## Future challenges and opportunities

### Challenges and solutions

A defining challenge for the WDR protein family is that most members act as β-propeller interaction platforms that nucleate multi-protein assemblies rather than catalyze reactions through a deep active site. Accordingly, disease-relevant phenotypes commonly arise from protein–protein interaction (PPI) topology, complex composition, and partner-dependent conformational states, which makes conventional “active-site inhibitor” paradigms less effective and increases the risk of shallow, context-sensitive chemical matter [20]. A second obstacle is strong context dependence across lineages and molecular states: the same WDR node can appear oncogenic in one setting yet neutral or even protective in another, because network wiring, redundancy, and pathway compensation differ by tissue, genotype, and treatment pressure [15]. Third, selectivity and tolerability can be difficult to balance: WD40 folds are widespread and frequently embedded in essential chromatin, RNA, and ubiquitin systems, so perturbing scaffolding functions can produce mechanism-linked liabilities; meanwhile, tumors can adapt by rewiring interactomes (partner switching or bypass activation), limiting response durability [366].

**Fig. 6:**
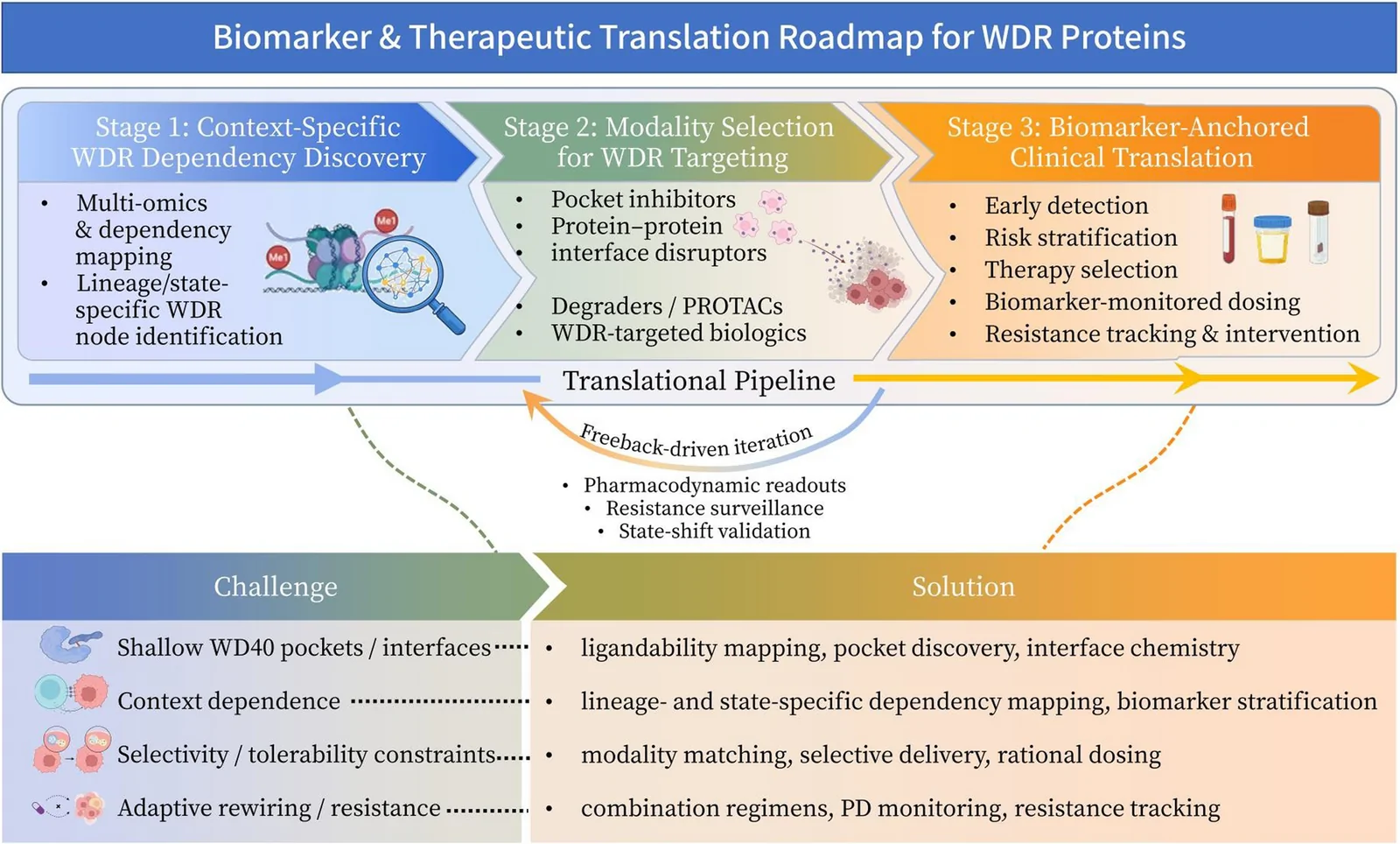
Biomarker and therapeutic translation roadmap for WDR proteins in cancer. This figure outlines a conceptual roadmap for translating WD40-repeat (WDR) proteins from mechanistic discoveries to biomarker-guided clinical applications

A practical way forward is to treat WDR proteins as a target class and deploy family-wide ligandability pipelines that integrate protein production, structure determination, and orthogonal biophysical/cellular assays to identify truly tractable pockets early, reducing late-stage attrition. A recent study exemplifies this approach by establishing a systematic workflow to scale discovery and characterization of small-molecule ligands across the WDR family [3]. In parallel, two modality shifts are expanding translational feasibility beyond “direct PPI blockade.” First, molecular-glue pharmacology leverages WD40 adaptors in ubiquitin ligase systems to create neomorphic interfaces that drive selective substrate loss; importantly, the sulfonamide E7820 recruits the CRL4 substrate receptor DCAF15 to promote RBM39 degradation, and a phase II clinical trial reported patient-level evidence of on-mechanism RBM39 degradation, supporting induced-proximity as a clinically actionable route for WD40 biology [367]. Second, targeted protein degradation (PROTACs/glue-derived degraders) can be especially advantageous for scaffold-like WDR proteins where partial interface inhibition may not extinguish function; by eliminating the scaffold, degradation can collapse multiple outputs and mitigate compensation. Recent syntheses around the PRC2 WD40 subunit EED summarize allosteric inhibitors, PPI-directed agents, and PROTAC strategies, while emphasizing pharmacodynamic frameworks that directly measure engagement, complex remodeling, and downstream state change [368] (Fig. 6).

### Future opportunities

#### Potential of WDR proteins as tumor biomarkers

The WDR protein family is one of the largest superfamilies in the human proteome, functioning as scaffolds for protein–protein interactions involved in signaling transduction, transcriptional regulation, cell cycle control, and other key biological processes. WDR proteins are extensively implicated in the pathogenesis of various cancers [349]. As such, WDR proteins have garnered increasing attention in oncology, demonstrating great potential as novel cancer biomarkers. Mounting evidence indicates that aberrant expression or genetic alterations of WDR proteins in different tumor types are closely associated with disease progression and patient outcomes. For example, WDR54 is highly expressed in CRC and is an independent prognostic factor associated with shorter disease-free survival [148]; in low-grade gliomas, overexpression of WDR76 correlates with more aggressive molecular phenotypes and independently predicts poorer prognosis [12]. Similarly, WDR72 is upregulated in NSCLC [369], and its expression is significantly correlated with prognosis and immune microenvironment indicators, supporting its role as a promising prognostic marker in lung cancer.

Importantly, the biomarker value of WDR proteins extends beyond prognosis to early detection and therapeutic response prediction. Alterations in certain WDR family members may occur at early stages of tumorigenesis, offering opportunities for early cancer diagnosis. For instance, WDR43 is markedly upregulated in CRC and functionally promotes tumor development, suggesting its potential as a diagnostic and therapeutic biomarker candidate [204]. Additionally, mutations in WDR genes may influence treatment response. A recent study reported that NSCLC patients harboring WDR49 mutations exhibit better response rates and survival benefits from immune checkpoint inhibitor therapy, indicating that WDR49 mutations could serve as predictive biomarkers for immunotherapy efficacy [370]. In the future, large-scale prospective clinical studies are warranted to validate the reliability of such markers and to integrate WDR protein-related biomarkers into early screening, prognostic models, and response monitoring, thereby advancing early diagnosis, accurate prognostication, and personalized treatment guidance in cancer care.

#### Clinical feasibility and limitations of WDR-based predictive biomarkers

Although WDR proteins are increasingly proposed as biomarkers for prognosis, immunotherapy response, and targeted therapy selection, their clinical utility should be evaluated cautiously. At present, most WDR-related biomarker evidence remains association-based rather than decision-grade [371, 372]. A major limitation is specificity. Many WDR proteins are broadly expressed and participate in fundamental processes such as chromatin regulation, ubiquitin-dependent proteostasis, ribosome biogenesis, RNA processing, and cytoskeletal remodeling [3]. Therefore, altered expression of a single WDR protein may reflect general proliferative activity, cellular stress, or lineage identity rather than a tumor-specific dependency. This issue is particularly relevant for WDR5 [72], RBBP4/7 [167], DDB1, and several DCAF proteins [373], whose biological functions are not restricted to malignant cells. For this reason, WDR proteins are unlikely to serve as robust stand-alone biomarkers in most clinical settings. Their specificity may be improved when they are incorporated into tumor-type-specific signatures, interpreted together with genomic context, pathway activity, immune infiltration features, and established biomarkers such as PD-L1 expression, tumor mutational burden [374], MSI status [375], driver mutations [376], or PRC2-related dependency markers [65].

Sensitivity is another important concern. For predictive use, the key question is not simply whether a WDR protein is detectable, but whether its level or activity can reliably identify patients who will benefit from a given therapy [377]. WDR expression may be spatially heterogeneous within tumors and dynamically regulated by hypoxia, treatment pressure, inflammatory signals, and metabolic stress. As a result, a single pretreatment biopsy may not capture the full WDR-dependent state of a tumor. Moreover, mRNA abundance may not accurately reflect protein level, subcellular localization, post-translational modification, binding-partner availability, or complex assembly, all of which are central to WDR function [378]. For example, WDR5 dependency may be better captured by MYC-linked transcriptional output or chromatin engagement than by WDR5 expression alone [290], whereas EED inhibitor sensitivity may require evidence of PRC2 dependence, H3K27me3 maintenance, and an intact senescence or differentiation response rather than EED expression alone [65]. Similarly, WDR-related immune signatures may correlate with macrophage infiltration, checkpoint molecule expression, or T-cell exclusion, but such correlations do not necessarily prove that WDR status predicts checkpoint blockade benefit [379]. Therefore, WDR-based predictive signatures should ideally combine expression data with pathway-level, spatial, and functional readouts.

From a practical perspective, clinical implementation will depend heavily on assay format and standardization [380]. Immunohistochemistry is attractive because it is inexpensive, tissue-based, and compatible with routine pathology workflows, but its performance depends on antibody specificity, staining protocols, scoring systems, cutoff selection, and inter-observer reproducibility [381]. RNA-based panels may offer better multiplexing and quantitative reproducibility, but they require standardized normalization strategies, adequate tumor purity, and careful control of batch effects [382, 383]. Proteomic, phosphoproteomic, spatial transcriptomic, and single-cell approaches may more accurately capture WDR activity states, yet their cost, complexity, tissue requirements, and bioinformatic burden currently limit routine deployment [378]. Liquid biopsy strategies could eventually monitor WDR-associated resistance states longitudinally, but sensitivity may be insufficient in low-tumor-burden settings, and circulating signals may not distinguish tumor-intrinsic WDR dependency from microenvironmental or systemic responses [384, 385].

Accordingly, the most clinically realistic role for WDR-based biomarkers is not as universal single-gene tests, but as components of composite, context-aware signatures [203, 371]. For targeted therapy, such signatures should identify tumors in which a WDR protein is not only altered but functionally required, such as tumors with PRC2 dependence for EED-directed therapy [334], MYC or MLL-driven transcriptional addiction for WDR5-directed strategies [290], or defined ubiquitin-proteostasis rewiring for DCAF- or FBXW-related vulnerabilities [6]. For immunotherapy, WDR-based signatures should be evaluated as immune-state modifiers that complement, rather than replace, established predictors [386]. Future studies should therefore prioritize analytical validity, clinical validity, and clinical utility. This will require standardized assays, predefined cutoffs, multi-center retrospective validation, prospective biomarker-embedded trials, and longitudinal sampling to determine whether WDR signatures can predict response, monitor resistance, or guide rational combinations in real clinical decision-making.

#### Therapeutic targeting of WDR proteins

As central scaffolding molecules in multi-protein complexes, WDR proteins play critical roles in oncogenic signaling pathways. Their dysfunction often results in uncontrolled cell proliferation, evasion of apoptosis, and epigenetic dysregulation, which are hallmarks of cancer progression [2]. Although many WDR proteins lack enzymatic active sites and were once considered “undruggable,” their pivotal positions in tumorigenic networks make them highly attractive therapeutic targets [3]. In recent years, significant progress has been made in therapeutic targeting of WDR proteins. WDR5, a prototypical WDR family member, is an essential component of several oncogenic chromatin-modifying complexes and recruits oncogenic transcription factors such as MYC. It has thus emerged as a high-value anti-cancer target. Multiple selective WDR5 inhibitors and PROTAC-based degraders have shown promising preclinical efficacy [75]. With expanding understanding of their oncogenic functions, many WDR proteins are poised to become new focal points in targeted cancer therapy.

However, the same scaffolding property that makes WDR proteins attractive as regulatory nodes also creates a practical limitation for target selection. Many WD40 proteins operate in paralogous pairs, related subfamilies, or partially overlapping macromolecular modules. As a result, inhibition or depletion of one WDR member may not be sufficient to collapse the oncogenic program if another structurally or functionally related scaffold can maintain complex assembly, substrate recognition, or pathway output. This issue is particularly relevant for WDR proteins involved in chromatin regulation, autophagy, and ubiquitin-dependent proteostasis, where related β-propeller proteins may share binding partners or act on convergent biological processes. Representative examples include the histone-binding proteins RBBP4 and RBBP7 in chromatin-regulatory complexes [387], WDR45 and WDR45B in autophagy control [112], and β-TrCP1 and β-TrCP2 as WD40-containing F-box substrate receptors [388]. These examples do not imply that WDR proteins are poor therapeutic targets, but they indicate that the therapeutic value of an individual WDR protein depends on whether the tumor is truly dependent on that specific node rather than on a redundant scaffold module.

This consideration is important for clinical translation. Expression changes alone may overestimate the actionability of a WDR protein if functional dependency has not been demonstrated [352, 389]. Similarly, an interface-selective inhibitor may block only one interaction surface while leaving other scaffold functions or compensatory partners intact [339]. Functional redundancy may therefore contribute to weak single-agent activity, incomplete pathway suppression, or adaptive resistance during treatment [390]. Future WDR-targeted studies should move beyond target nomination based on expression or mutation status and incorporate paralog-aware dependency mapping, combinatorial perturbation screens, interface-specific rescue experiments, and longitudinal analysis of compensatory network rewiring [391]. In tumors where a WDR protein controls a dominant, non-redundant structural pocket or disease-critical complex, selective inhibition remains a rational strategy. In settings where redundancy is prominent, targeted degradation, dual-interface blockade, or combination therapy against downstream pathway outputs may provide a more realistic route to durable therapeutic benefit.

Innovative precision-targeting strategies have opened new opportunities for drug development against WDR proteins, particularly when tumor dependency and druggable interfaces can be clearly defined. Among these approaches, small-molecule inhibitors have been developed to disrupt oncogenic interaction interfaces mediated by WDR proteins. WDR5 is the most representative example. Several WDR5-directed inhibitors targeting its protein-interaction surfaces have shown strong anti-tumor activity in preclinical models by interfering with oncogenic chromatin programs. In parallel, PROTAC-based strategies provide a complementary route for WDR targeting by eliminating the scaffold protein itself rather than blocking only one interaction interface. Selective WDR5 degraders, such as MS33 and MS67, recruit E3 ubiquitin ligases to induce WDR5 degradation and have been shown to suppress leukemia progression in xenograft models and prolong survival [165]. Peptide-based or biologic strategies that disrupt WDR-mediated protein–protein interactions are also being explored. For example, short peptides targeting the WDR5-MLL1 interaction interface can interfere with complex formation, restore histone modification balance, and inhibit leukemia cell proliferation [76]. These studies indicate that historically “undruggable” WDR scaffolds can become therapeutically accessible when disease-relevant interfaces, degradation strategies, or dependency contexts are precisely defined.

Combining WDR-directed strategies with immunotherapy may represent another important direction. Several WDR proteins have been implicated in immune evasion and tumor microenvironment remodeling, suggesting that their targeting could influence anti-tumor immunity in selected contexts. For instance, WDR76 overexpression in glioma has been associated with immunosuppressive M2 macrophage infiltration and increased expression of immune checkpoint-related molecules, supporting its potential relevance to immunotherapy-oriented stratification [12]. Future studies should further define the immunomodulatory roles of WDR proteins and explore rational combinations with immune checkpoint blockade, epigenetic therapy, targeted degraders, or nanoparticle-based delivery systems. In summary, WDR proteins are expected to occupy an increasingly important position in targeted cancer therapy, but their clinical translation will require careful distinction between broadly altered WDR expression and true tumor-state-specific dependency. As more oncogenic WDR targets, compensatory scaffold networks, and druggable interaction interfaces are defined, WDR-directed strategies may expand current treatment options and support more precise combination therapies for selected patient populations.

#### Artificial intelligence and machine learning-assisted WDR research

Artificial intelligence and machine learning are emerging as important accelerators of WDR protein research, particularly because most WDR proteins function through structural interfaces, protein–protein interactions, and context-dependent scaffold assemblies rather than through classical catalytic active sites. AlphaFold has markedly expanded the structural coverage of the human proteome and provides a practical starting point for annotating poorly characterized WD40 β-propellers, predicting blade organization, and identifying candidate pockets, rim-loop interfaces, or partner-binding surfaces for experimental validation [392, 393]. This is especially relevant for WDR proteins, because WD40 repeats are often sequence-degenerate, and their oncogenic functions depend more on three-dimensional interaction topology than on primary sequence conservation alone. More recent complex-prediction frameworks, including AlphaFold 3, further extend this opportunity by modeling biomolecular complexes containing proteins, nucleic acids, small molecules, ions, and modified residues, thereby providing a useful platform for generating hypotheses about WDR-partner recognition, WDR-RNA interactions, and ligandable interface discovery [394]. AI-assisted structural biology can also be integrated with large-scale interactomics to define WDR-dependent regulatory networks. AlphaFold-based modeling has already been used to move toward a structurally resolved human protein interaction network, and newer resources such as hu.MAP3.0 use machine learning to integrate high-throughput proteomic datasets into more comprehensive maps of human protein complexes [395, 396]. For WDR proteins, such strategies could help distinguish stable core complex components from transient, context-specific, or treatment-induced partners. This distinction is important because the same WDR protein may support different oncogenic outputs depending on lineage, genotype, post-translational modification state, subcellular localization, or therapeutic pressure. In practice, combining structural prediction, proximity labeling, affinity purification mass spectrometry, phosphoproteomics, and perturbation screening may enable a more accurate definition of WDR-centered dependencies than transcriptomic profiling alone [349, 397]. AI and machine learning may further accelerate WDR-targeted drug discovery. Because many WDR proteins are scaffold-like and historically difficult to drug, computational models can be used to rank ligandable pockets, evaluate interface selectivity, perform virtual screening, and guide the design of small molecules, molecular glues, or targeted degraders against disease-critical WDR complexes. A recent AI-guided pipeline for protein–protein interaction drug discovery combined quantitative interaction assays, machine learning-based scoring, AlphaFold-Multimer modeling, and experimental validation to prioritize druggable PPIs, providing a methodological template that is conceptually well suited to WDR proteins [398]. In the WDR field, this approach could help separate proteins with dominant, druggable pockets, such as EED- or WDR5-like paradigms, from multi-interface scaffolds that may require targeted degradation, dual-interface blockade, or rational combination therapy.

Beyond target discovery, machine learning may improve patient stratification by integrating WDR-centered multi-omics data with clinical and pathological variables. Instead of relying on single WDR expression values, future models could combine genomics, transcriptomics, proteomics, phosphoproteomics, spatial profiling, immune infiltration features, treatment history, and pharmacodynamic readouts to define WDR-dependent tumor states. Recent reviews of AI-driven multi-omics integration in precision oncology emphasize its potential for biomarker discovery, diagnosis, prognosis prediction, therapy response modeling, and clinical decision support [399, 400]. For WDR-based oncology, such models may be particularly useful for identifying tumors with PRC2 dependence for EED-directed therapy, MYC- or MLL-linked transcriptional addiction for WDR5-targeted strategies, or ubiquitin-proteostasis rewiring involving DCAF- or FBXW-related vulnerabilities.

## Conclusion

WDR proteins comprise one of the largest eukaryotic protein families and typically fold into β-propeller platforms optimized for protein–protein interactions, positioning them as higher-order organizers of transcription, epigenetic regulation, ubiquitination, and cell-cycle control. Across cancers, the central theme that emerges from this review is that WDR proteins act less like single “pathway switches” and more like state-shaping scaffolds that coordinate complex composition, spatial proximity, and signal integration across chromatin, RNA/translation, proteostasis, cytoskeletal remodeling, ribosome biogenesis, and genome maintenance. This systems role helps explain their pervasive, context-dependent contributions to malignant phenotypes, and it also provides a coherent framework to interpret cross-tumor differences as shifts in which WDR-governed layer most tightly couples to the dominant clinical bottleneck of a given disease setting.

From a translational perspective, WDR proteins are moving from “biologically important but pharmacologically difficult” to actionable targets. The most mature clinical foothold is achieved where a structurally druggable WD40 pocket controls a disease-critical epigenetic switch, exemplified by EED inhibitors that have entered early-phase oncology trials, establishing human feasibility for WD40-pocket pharmacology. In parallel, preclinical innovation is broadening modality space for scaffold proteins, including interface-disrupting small molecules, PROTAC degraders, and emerging biologics designed to collapse oncogenic assemblies rather than inhibit catalytic active sites. Importantly, the review also highlights a practical opportunity to connect WDR targeting to immuno-oncology, because several WDR nodes intersect immune evasion and microenvironmental remodeling, making rational combination strategies with checkpoint blockade an increasingly testable direction.

Clinically, the biomarker promise of WDR proteins extends beyond prognosis toward earlier detection and treatment selection, including potential predictive links to immunotherapy benefit in defined genomic contexts; however, these signals now require rigorous prospective validation and harmonized assay/threshold frameworks before they can be integrated into decision-making. Looking forward, the most impactful progress will depend on integrating tumor-type and state-resolved dependency mapping with structure-guided modality selection and biomarker-anchored clinical testing. Multi-omics coupled to functional perturbation can define which WDR nodes are truly required in a given lineage, genotype, and treatment state; structural and chemical biology can then determine how best to perturb scaffold function with sufficient selectivity, whether by disrupting key interfaces or dismantling entire assemblies; and trial designs that embed pharmacodynamic and state-shift readouts can establish when WDR modulation meaningfully reprograms tumor biology and improves outcomes, particularly in rational combination regimens. Executed as an iterative, feedback-driven pipeline, this framework can move the WDR protein family beyond a catalog of mechanisms toward a clinically enabling layer of state-aligned biomarkers and actionable therapeutic vulnerabilities across diverse cancers.

## Supplementary Information

Supplementary Material 1.

## Data Availability

No datasets were generated or analysed during the current study.

## Abbreviations

WDR: WD40-repeat
EC: Esophageal cancer
ESCC: Esophageal squamous cell carcinoma
GC: Gastric cancer
HCC: Hepatocellular carcinoma
PDAC: Pancreatic ductal adenocarcinoma
CRC: Colorectal cancer
BC: Breast cancer
OC: Ovarian cancer
CC: Cervical cancer
RCC: Renal cell carcinoma
ccRCC: Clear cell Renal cell carcinoma
BCa: Bladder cancer
